# Supplementary material for: Unveiling gene perturbation effects through gene regulatory networks inference from single-cell transcriptomic data
Source: PLoS Comput Biol. 2026 Apr 15;22(4):e1014067. doi: 10.1371/journal.pcbi.1014067 (PMC13082667; doi:10.1371/journal.pcbi.1014067)
Supplement: S1 Fig — (PDF) [file pcbi.1014067.s001.pdf]

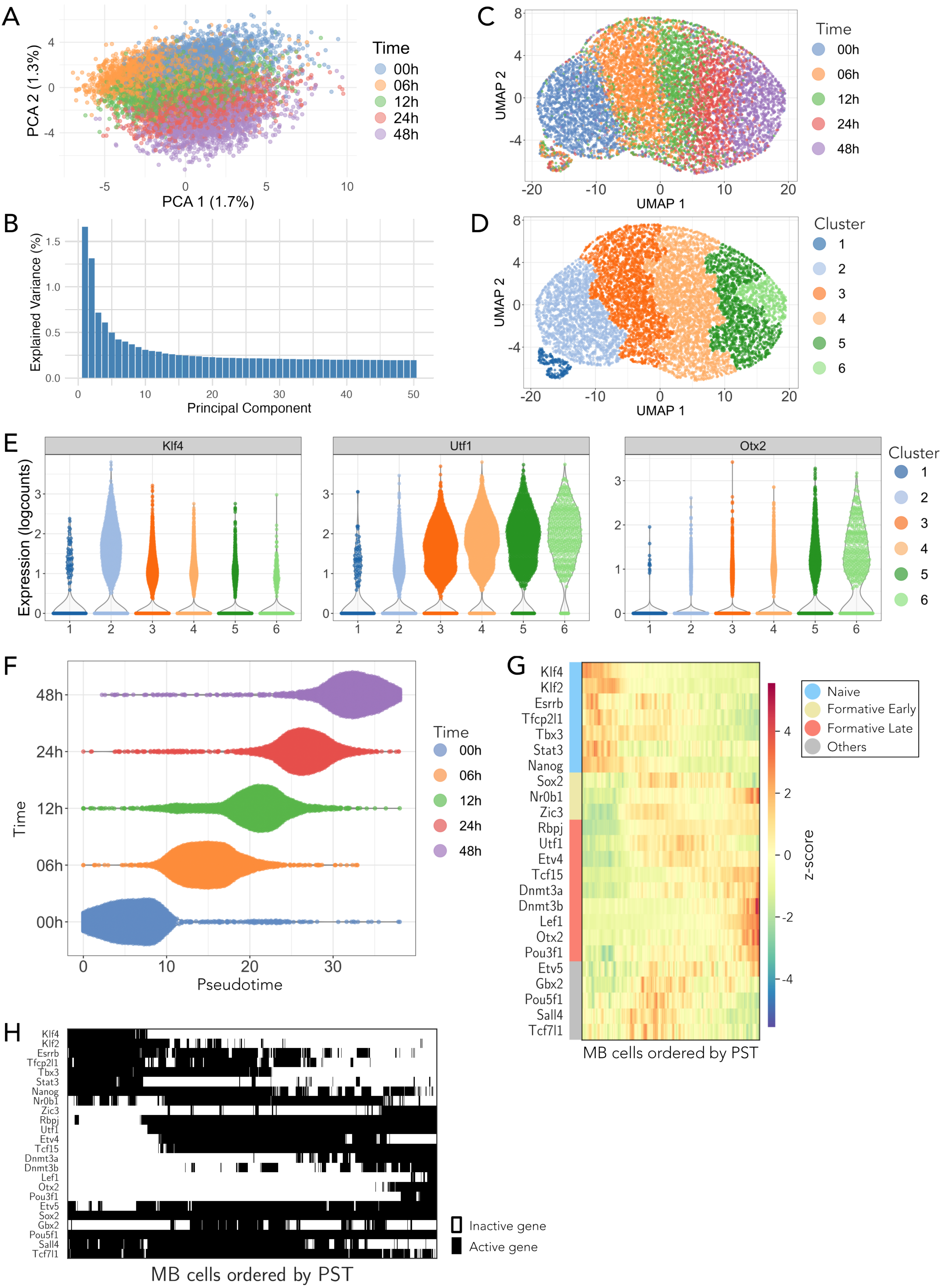

A
B
C
D
E
F
G
H
PCA 1 (1.7%)
PCA 2 (1.3%)
Time
00h
06h
12h
24h
48h
Explained Variance (%)
Principal Component
UMAP 1
UMAP 2
Cluster
Klf4
Utf1
Otx2
Expression (logcounts)
Pseudotime
Naive
Formative Early
Formative Late
Others
z-score
MB cells ordered by PST
Inactive gene
Active gene
Klf4
Klf2
Esrrb
Tfcp2l1
Tbx3
Stat3
Nanog
Sox2
Nr0b1
Zic3
Rbpj
Utf1
Etv4
Tcf15
Dnmt3a
Dnmt3b
Lef1
Otx2
Pou3f1
Etv5
Gbx2
Pou5f1
Sall4
Tcf7l1

S1 Figure. Preservation of gene expression dynamics following pseudotime and Mini-bulk processing.

A. Two-dimensional PCA of the input experimental dataset (scRNA-seq data with LogNorm), computed on the 2318 selected genes. Each point represents an individual cell coloured by sampling time.

B. Percentage of variance explained by the first 50 principal components of the input experimental dataset.

C. UMAP of the input experimental dataset (scRNA-seq data with LogNorm). Each point represents an individual cell coloured by sampling time.

D. UMAP of the same dataset as in A, but with cells coloured by clustering label.

E. Gene expression levels across the six identified clusters for three representative genes (Klf4, Utf1, and Otx2). Each violin plot shows the distribution of log-normalized expression values, with the width reflecting the density of cells at different expression levels.

F. Distribution of pseudotime values across sampling time points (0h, 6h, 12h, 24h, 48h). Each violin corresponds to one time point, with the width reflecting the density of cells at each pseudotime interval.

G. Gene expression z-scores for the input dataset (scRNA-seq with LogNorm), with cells ordered by pseudotime (PST) and grouped into Mini-Bulk (MB) bins. Genes are coloured according to their group as in Fig. 1C (naïve, formative early, formative late, others).

H. Gene activity for the same dataset, binarised as active (black) or inactive (white) using a threshold at half the maximum expression for each gene. Rows correspond to genes, columns to cells ordered by pseudotime and grouped into Mini-Bulk cells.
