# Supplementary material for: Unveiling gene perturbation effects through gene regulatory networks inference from single-cell transcriptomic data
Source: PLoS Comput Biol. 2026 Apr 15;22(4):e1014067. doi: 10.1371/journal.pcbi.1014067 (PMC13082667; doi:10.1371/journal.pcbi.1014067)
Supplement: S2 Fig — (PDF) [file pcbi.1014067.s002.pdf]

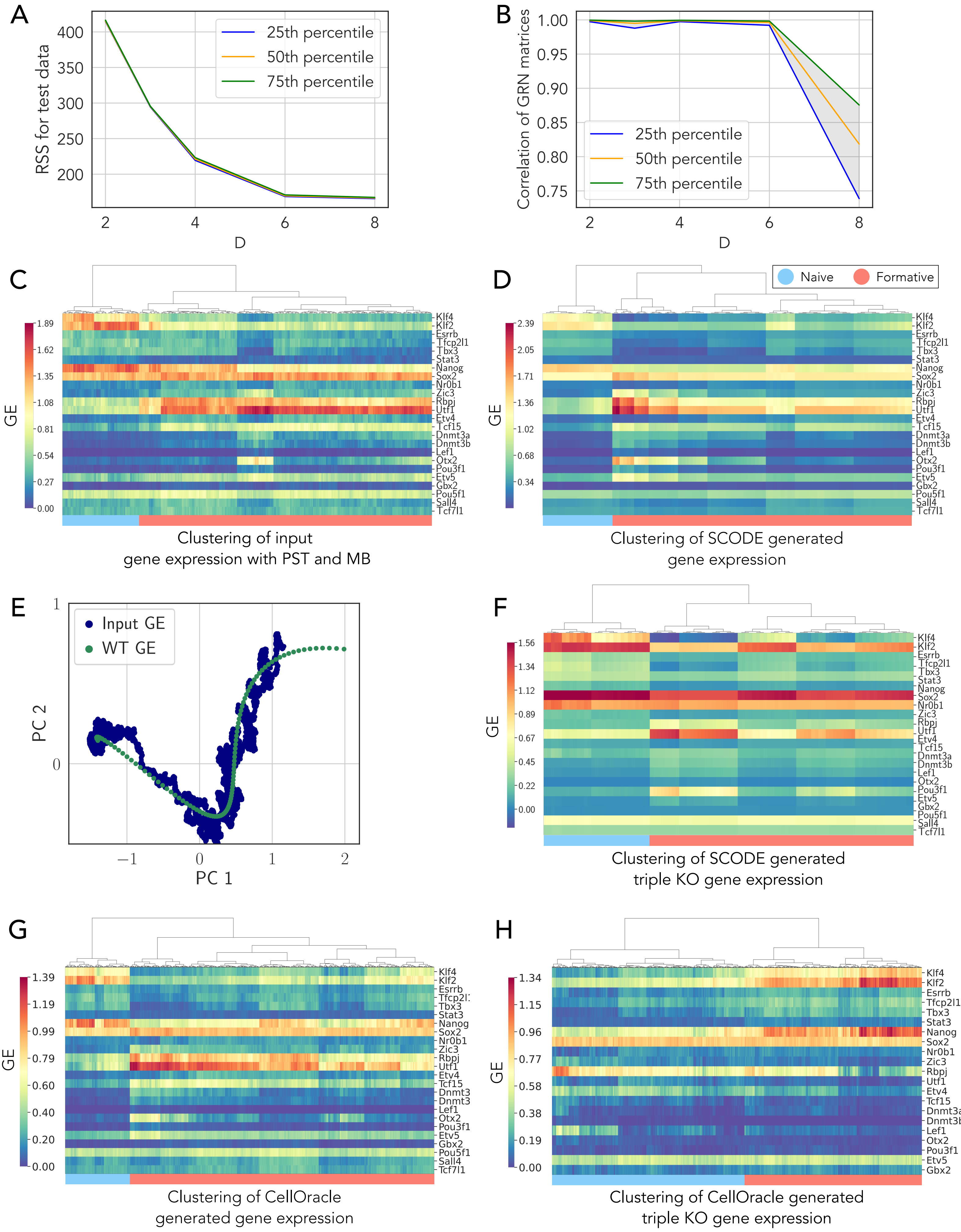
A
RSS for test data
400
350
300
250
200
2
4
6
8
D
25th percentile
50th percentile
75th percentile
B
Correlation of GRN matrices
1.00
0.95
0.90
0.85
0.80
0.75
2
4
6
8
D
25th percentile
50th percentile
75th percentile
C
GE
1.89
1.62
1.35
1.08
0.81
0.54
0.27
0.00
Klf4
Klf2
Esrrb
Tfcp2l1
Tbx3
Stat3
Nanog
Sox2
Nr0b1
Zic3
Rbpj
Utf1
Etv4
Tcf15
Dnmt3a
Dnmt3b
Lef1
Otx2
Pou3f1
Etv5
Gbx2
Pou5f1
Sall4
Tcf7l1
Clustering of input
gene expression with PST and MB
D
Naive
Formative
GE
2.39
2.05
1.71
1.36
1.02
0.68
0.34
Clustering of SCODE generated
gene expression
E
Input GE
WT GE
PC 2
PC 1
1
0
−1
0
1
2
F
GE
1.56
1.34
1.12
0.89
0.67
0.45
0.22
0.00
Clustering of SCODE generated
triple KO gene expression
G
GE
1.39
1.19
0.99
0.79
0.60
0.40
0.20
0.00
Clustering of CellOracle
generated gene expression
H
GE
1.34
1.15
0.96
0.77
0.58
0.38
0.19
0.00
Clustering of CellOracle generated
triple KO gene expression

S2 Figure. SCODE parameter optimization and validation of SCODE and CellOracle generated data against input mouse scRNA-seq.

A. Residual sum of squares (RSS) values of the test data across different values of D (dimensionality of the reduced expression dynamics representation) in SCODE analysis. Curves show the 25th, 50th, and 75th percentiles.

B. Correlation of inferred GRN matrices across 50 replicates (in ascending order of RSS values) for different values of D in SCODE analysis. Curves show the 25th, 50th, and 75th percentiles.

C. Gene expression of the input dataset (scRNA-seq data with LogNorm) after pseudotime (PST) ordering and Mini-Bulk (MB) grouping. Rows correspond to genes and columns to MB cells; hierarchical clustering was applied to genes. The dataset has 9547 cells.

D. Gene expression of SCODE-generated data. Rows correspond to genes and columns to simulated cells; hierarchical clustering was applied to genes. 100 cells were simulated, as suggested by the authors of SCODE [1].

E. PCA of gene expression for the input dataset and SCODE-generated wild-type (WT) data. Each point corresponds to one cell.

F. Gene expression of SCODE-generated triple KO data. Rows correspond to genes and columns to simulated cells; hierarchical clustering was applied to genes. 100 cells were simulated, as in WT state.

G. Gene expression of CellOracle-generated data. Rows correspond to genes and columns to simulated cells; hierarchical clustering was applied to genes. The dataset has 9547 cells, as the input one.

H. Gene expression of CellOracle-generated triple KO data. Rows correspond to genes and columns to simulated cells; hierarchical clustering was applied to genes. The dataset has 9547 cells, as the input one.

References

[1] Hirotaka Matsumoto et al. "SCODE: an efficient regulatory network inference algorithm from single-cell RNA-Seq during differentiation". In: *Bioinformatics* 33.15 (Apr. 2017), pp. 2314–2321. ISSN: 1367-4803. DOI: 10.1093/bioinformatics/btx194. eprint: https://academic.oup.com/bioinformatics/article-pdf/33/15/2314/50756465/bioinformatics_33_15_2314.pdf. URL: https://doi.org/10.1093/bioinformatics/btx194.
