# Supplementary material for: Unveiling gene perturbation effects through gene regulatory networks inference from single-cell transcriptomic data
Source: PLoS Comput Biol. 2026 Apr 15;22(4):e1014067. doi: 10.1371/journal.pcbi.1014067 (PMC13082667; doi:10.1371/journal.pcbi.1014067)
Supplement: S3 Fig — (PDF) [file pcbi.1014067.s003.pdf]

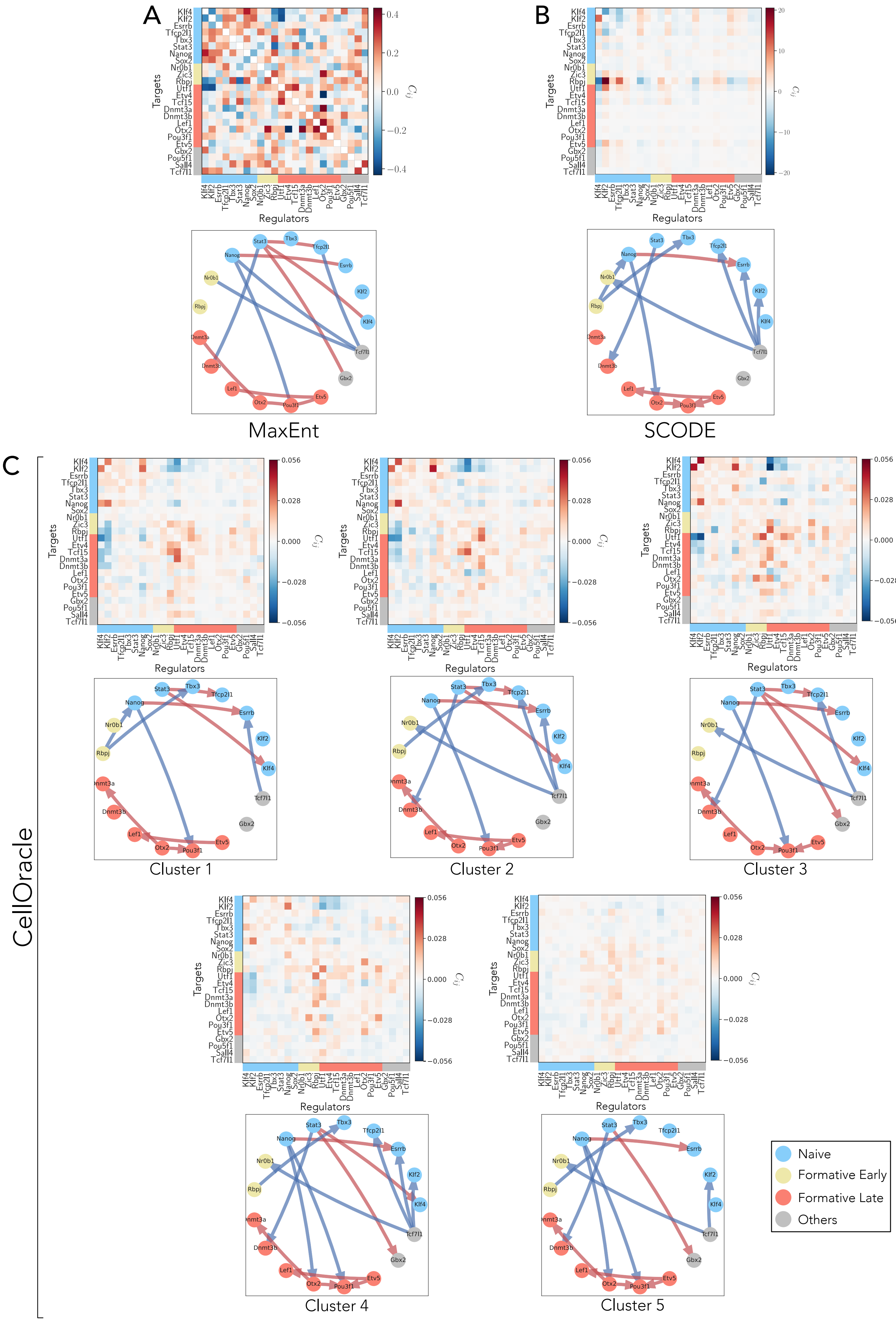
A
B
C
Klf4
Klf2
Esrrb
Tfcp2l1
Tbx3
Stat3
Nanog
Sox2
Nr0b1
Zic3
Rbpj
Utf1
Etv4
Tcf15
Dnmt3a
Dnmt3b
Lef1
Otx2
Pou3f1
Etv5
Gbx2
Pou5f1
Sall4
Tcf7l1
Targets
Regulators
C_{ij}
0.4
0.2
0.0
−0.2
−0.4
20
10
0
−10
−20
0.056
0.028
0.000
−0.028
−0.056
MaxEnt
SCODE
CellOracle
Cluster 1
Cluster 2
Cluster 3
Cluster 4
Cluster 5
Naive
Formative Early
Formative Late
Others

S3 Figure. GRNs inferred with alternative methods on the mouse dataset.

A. MaxEnt-inferred undirected GRN from the input dataset (scRNA-seq data with LogNorm, PST, and MB). Upper: interaction matrix between regulators (columns) and targets (rows), presented in a style analogous to Fig. 4A. Lower: network representation of literature-supported interactions correctly inferred by MaxEnt, presented in a style analogous to Fig. 4C.

B. SCODE-inferred GRN from the input dataset. Upper: interaction matrix between regulators (columns) and targets (rows). Lower: network representation of literature-supported interactions correctly inferred by SCODE.

C. CellOracle-inferred GRNs from the input dataset for each cluster (1–5). Upper: interaction matrices between regulators (columns) and targets (rows). Lower: network representation of literature-supported interactions correctly inferred by CellOracle. Colours of nodes indicate gene groups as in Fig. 1C.
