# Supplementary material for: Unveiling gene perturbation effects through gene regulatory networks inference from single-cell transcriptomic data
Source: PLoS Comput Biol. 2026 Apr 15;22(4):e1014067. doi: 10.1371/journal.pcbi.1014067 (PMC13082667; doi:10.1371/journal.pcbi.1014067)
Supplement: S4 Fig — (PDF) [file pcbi.1014067.s004.pdf]

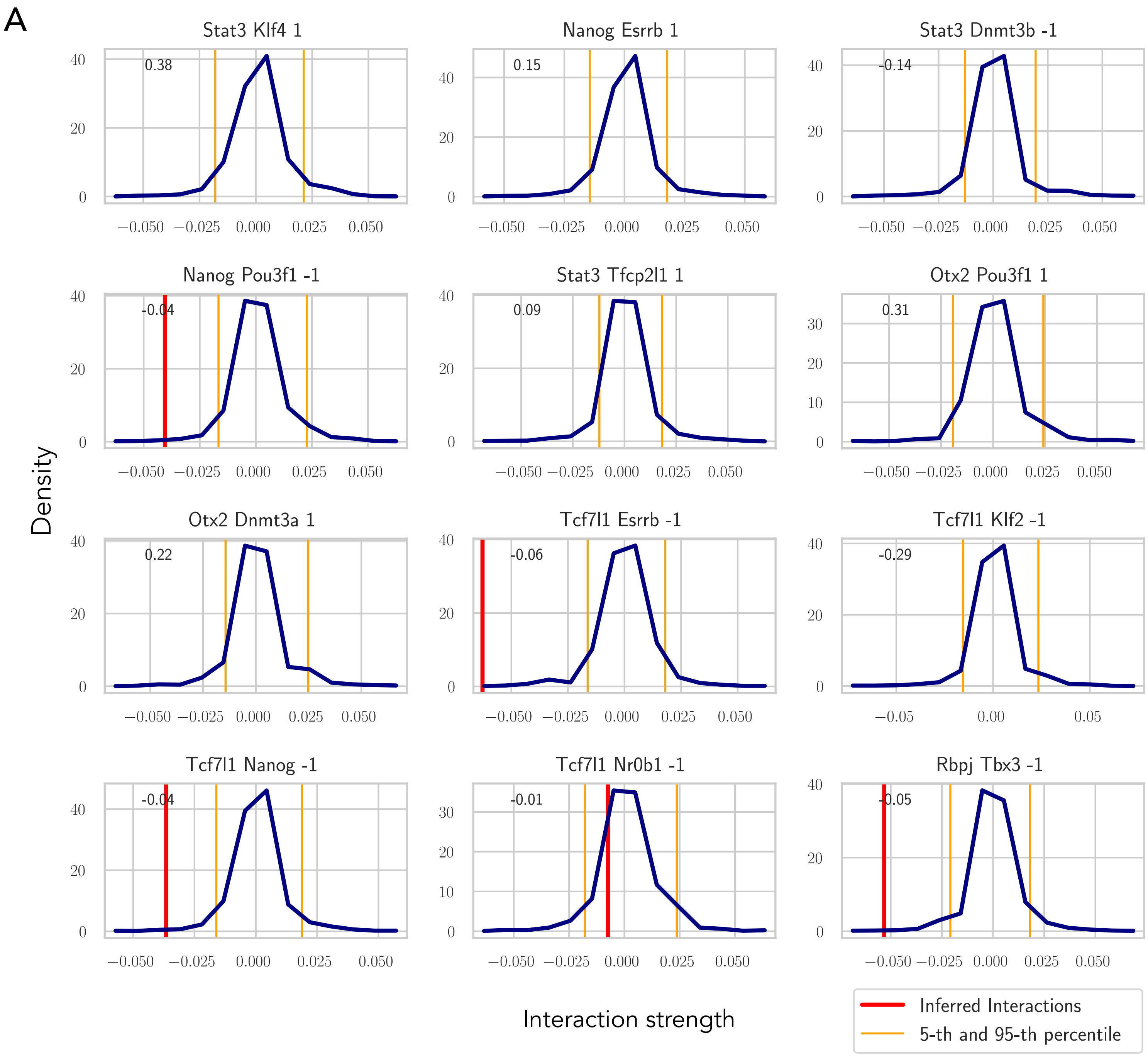
A
Stat3 Klf4 1
0.38
Nanog Esrrb 1
0.15
Stat3 Dnmt3b -1
-0.14
Nanog Pou3f1 -1
-0.04
Stat3 Tfcp2l1 1
0.09
Otx2 Pou3f1 1
0.31
Otx2 Dnmt3a 1
0.22
Tcf7l1 Esrrb -1
-0.06
Tcf7l1 Klf2 -1
-0.29
Tcf7l1 Nanog -1
-0.04
Tcf7l1 Nr0b1 -1
-0.01
Rbpj Tbx3 -1
-0.05
Density
Interaction strength
Inferred Interactions
5-th and 95-th percentile

S4 Figure. Statistical validation of inferred interactions against a null model.

A. Distribution of interaction values for selected pairs of genes from 2500 GRNs. These networks are inferred with IGNITE using 50 shuffled datasets as input. These datasets are randomly shuffled versions of the input dataset (scRNA-seq data with LogNorm, PST, and MB). Then for each of these datasets 50 sets of hyperparameters were used (see Methods for details). Each panel illustrates the distribution of specific gene pair interaction values across the IGNITE-derived GRNs (with $N_{bins} = 15$). The red lines indicate the interaction values from the IGNITE GRN with the lowest CMD, while the yellow lines delineate the fifth and 95th percentile confidence intervals, providing a basis for evaluating the significance of each interaction.
