# Supplementary material for: Unveiling gene perturbation effects through gene regulatory networks inference from single-cell transcriptomic data
Source: PLoS Comput Biol. 2026 Apr 15;22(4):e1014067. doi: 10.1371/journal.pcbi.1014067 (PMC13082667; doi:10.1371/journal.pcbi.1014067)
Supplement: S5 Fig — (PDF) [file pcbi.1014067.s005.pdf]

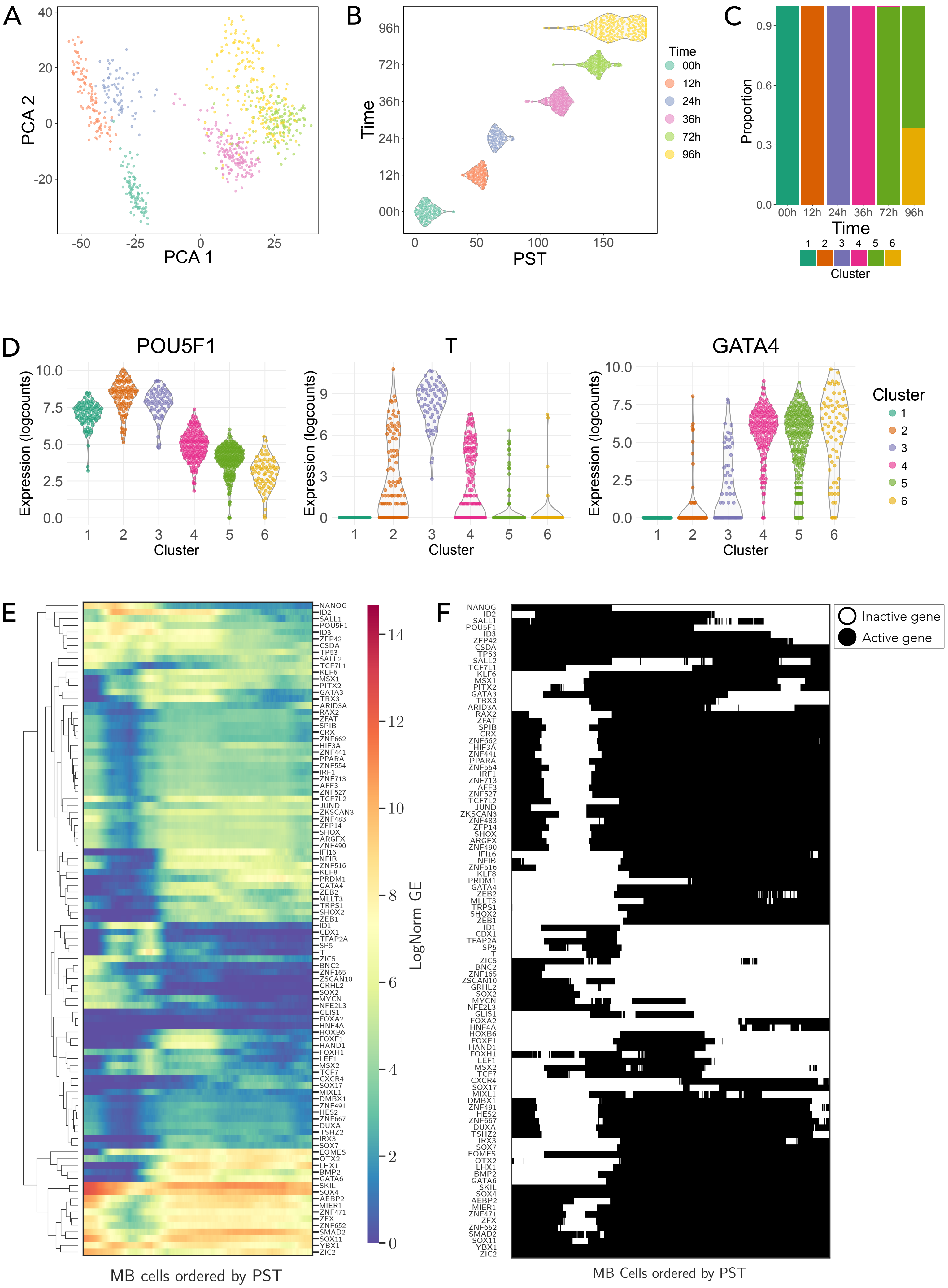
A
PCA 1
PCA 2
B
Time
PST
Time
00h
12h
24h
36h
72h
96h
C
Proportion
Time
Cluster
D
POU5F1
T
GATA4
Expression (logcounts)
Cluster
E
LogNorm GE
MB cells ordered by PST
NANOG ID2 SALL1 POU5F1 ID3 ZFP42 CSDA TP53 SALL2 TCF7L1 KLF6 MSX1 PITX2 GATA3 TBX3 ARID3A RAX2 ZFAT SPIB CRX ZNF662 HIF3A ZNF441 PPARA ZNF554 IRF1 ZNF713 AFF3 ZNF527 TCF7L2 JUND ZKSCAN3 ZNF483 ZFP14 SHOX ARGFX ZNF490 IFI16 NFIB ZNF516 KLF8 PRDM1 GATA4 ZEB2 MLLT3 TRPS1 SHOX2 ZEB1 ID1 CDX1 TFAP2A SP5 T ZIC5 BNC2 ZNF165 ZSCAN10 GRHL2 SOX2 MYCN NFE2L3 GLIS1 FOXA2 HNF4A HOXB6 FOXF1 HAND1 FOXH1 LEF1 MSX2 TCF7 CXCR4 SOX17 MIXL1 DMBX1 ZNF491 HES2 ZNF667 DUXA TSHZ2 IRX3 SOX7 EOMES OTX2 LHX1 BMP2 GATA6 SKIL SOX4 AEBP2 MIER1 ZNF471 ZFX ZNF652 SMAD2 SOX11 YBX1 ZIC2
F
Inactive gene
Active gene
MB Cells ordered by PST

S5 Figure. Gene expression patterns in human PSC differentiation.

A. PCA of the input dataset (scRNA-seq data with LogNorm). Each point represents an individual cell coloured by its sampling time (0h, 12h, 24h, 36h, 72h, 96h).

B. Pseudotime distributions across sampling times for the input dataset. Each violin corresponds to a time point, with colour indicating the sampling time. The width of each violin reflects the density of cells at each pseudotime value.

C. Proportion of cells from each cluster across sampling times. Bars indicate the relative fraction of cells belonging to the clusters (1–6) at each time point.

D. Expression of Pou5f1, T, and Gata4 across the six clusters of the input dataset. Violin plots show the distribution of log-normalized expression values, with width proportional to the density of cells at each level.

E. Gene expression (log-normalized, z-score) for the input dataset, ordered by pseudotime (PST) and Mini-Bulk (MB). Genes are arranged by hierarchical clustering to highlight expression patterns across differentiation.

F. Gene activity computed from the input dataset (log-normalization, PST and MB implementation), where genes are classified as active (black) or inactive (white) using a threshold at half of the maximum expression value per gene. Genes are ordered as in (E).
