# Supplementary material for: Unveiling gene perturbation effects through gene regulatory networks inference from single-cell transcriptomic data
Source: PLoS Comput Biol. 2026 Apr 15;22(4):e1014067. doi: 10.1371/journal.pcbi.1014067 (PMC13082667; doi:10.1371/journal.pcbi.1014067)
Supplement: S6 Fig — (PDF) [file pcbi.1014067.s006.pdf]

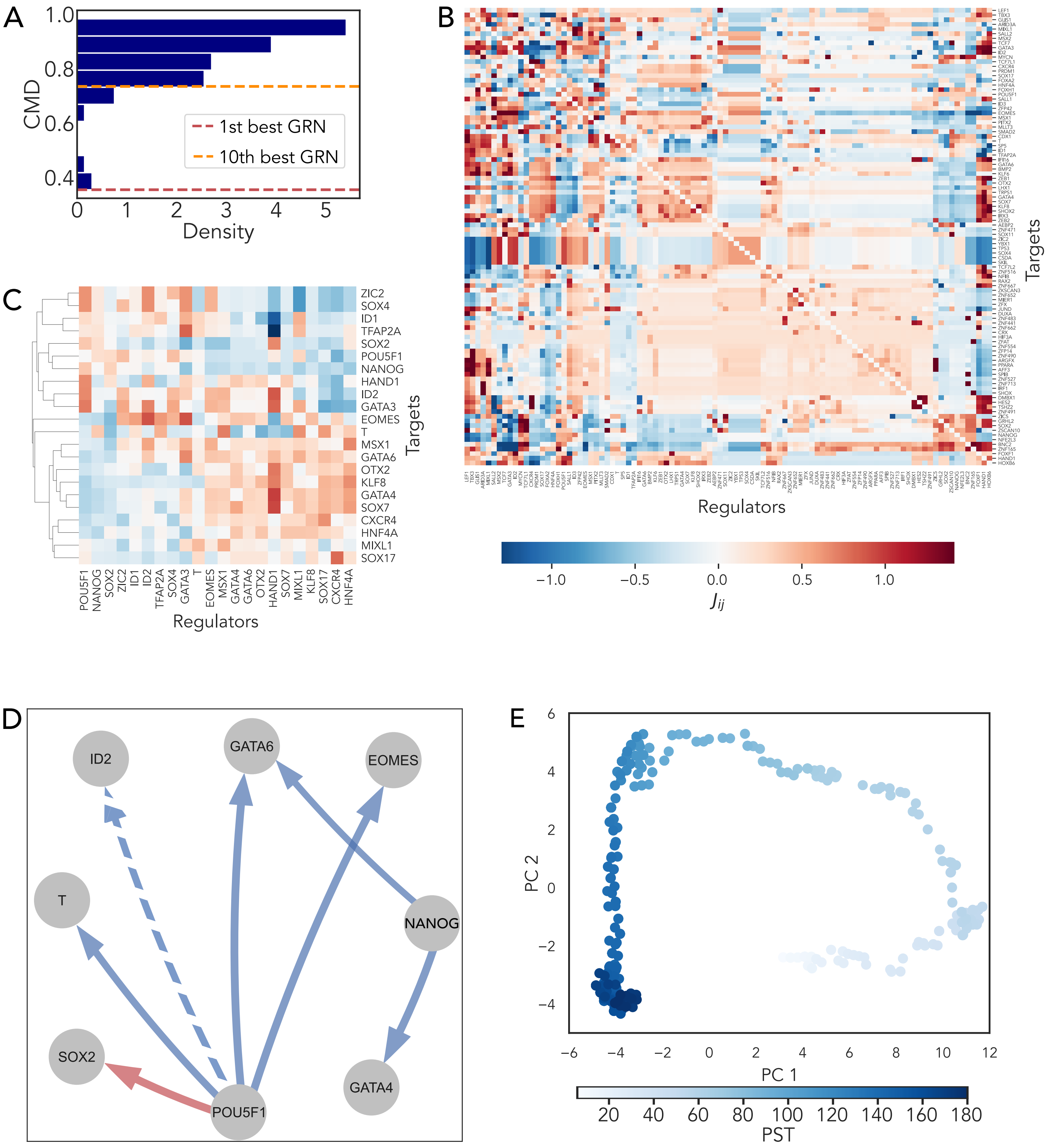
A
CMD
Density
1st best GRN
10th best GRN
B
Targets
Regulators
J_{ij}
C
ZIC2
SOX4
ID1
TFAP2A
SOX2
POU5F1
NANOG
HAND1
ID2
GATA3
EOMES
T
MSX1
GATA6
OTX2
KLF8
GATA4
SOX7
CXCR4
HNF4A
MIXL1
SOX17
Targets
POU5F1
NANOG
SOX2
ZIC2
ID1
ID2
TFAP2A
SOX4
GATA3
T
EOMES
MSX1
GATA4
GATA6
OTX2
HAND1
SOX7
MIXL1
KLF8
SOX17
CXCR4
HNF4A
Regulators
D
ID2
GATA6
EOMES
T
NANOG
SOX2
GATA4
POU5F1
E
PC 2
PC 1
PST

S6 Figure. Inference and selection of the best-performing human GRN.

A. Density distribution of Correlation Matrices Distance (CMD) values for the 150 inferred GRNs. The dashed lines indicate the CMD values of the best (lowest CMD) and the 10th best GRN.

B. IGNITE GRN interaction matrix inferred from the human input dataset (scRNA-seq data with LogNorm, PST, and MB). Each element (i, j) represents the interaction strength from regulator gene j to target gene i. Genes are ordered consistently using hierarchical clustering.

C. Subset of the IGNITE interaction matrix in (B), restricted to literature-reported genes involved in pluripotency and differentiation. Colours represent interaction strengths as in (B).

D. GRN reconstructed for the set of the interactions reported in [1], restricted to the genes included in our analysis. Arrows indicate direction and sign: activating (red), inhibiting (blue). Dashed lines represent interactions inferred incorrectly.

E. PCA of gene activity for the human input dataset (scRNA-seq data with LogNorm, PST, and MB). Each point corresponds to a cell, coloured by pseudotime (PST).

References

[1] Hirotaka Matsumoto et al. "SCODE: an efficient regulatory network inference algorithm from single-cell RNA-Seq during differentiation". In: *Bioinformatics* 33.15 (Apr. 2017), pp. 2314–2321. ISSN: 1367-4803. DOI: 10.1093/bioinformatics/btx194. eprint: https://academic.oup.com/bioinformatics/article-pdf/33/15/2314/50756465/bioinformatics_33_15_2314.pdf. URL: https://doi.org/10.1093/bioinformatics/btx194.
