# Supplementary material for: Unveiling gene perturbation effects through gene regulatory networks inference from single-cell transcriptomic data
Source: PLoS Comput Biol. 2026 Apr 15;22(4):e1014067. doi: 10.1371/journal.pcbi.1014067 (PMC13082667; doi:10.1371/journal.pcbi.1014067)
Supplement: S7 Fig — (PDF) [file pcbi.1014067.s007.pdf]

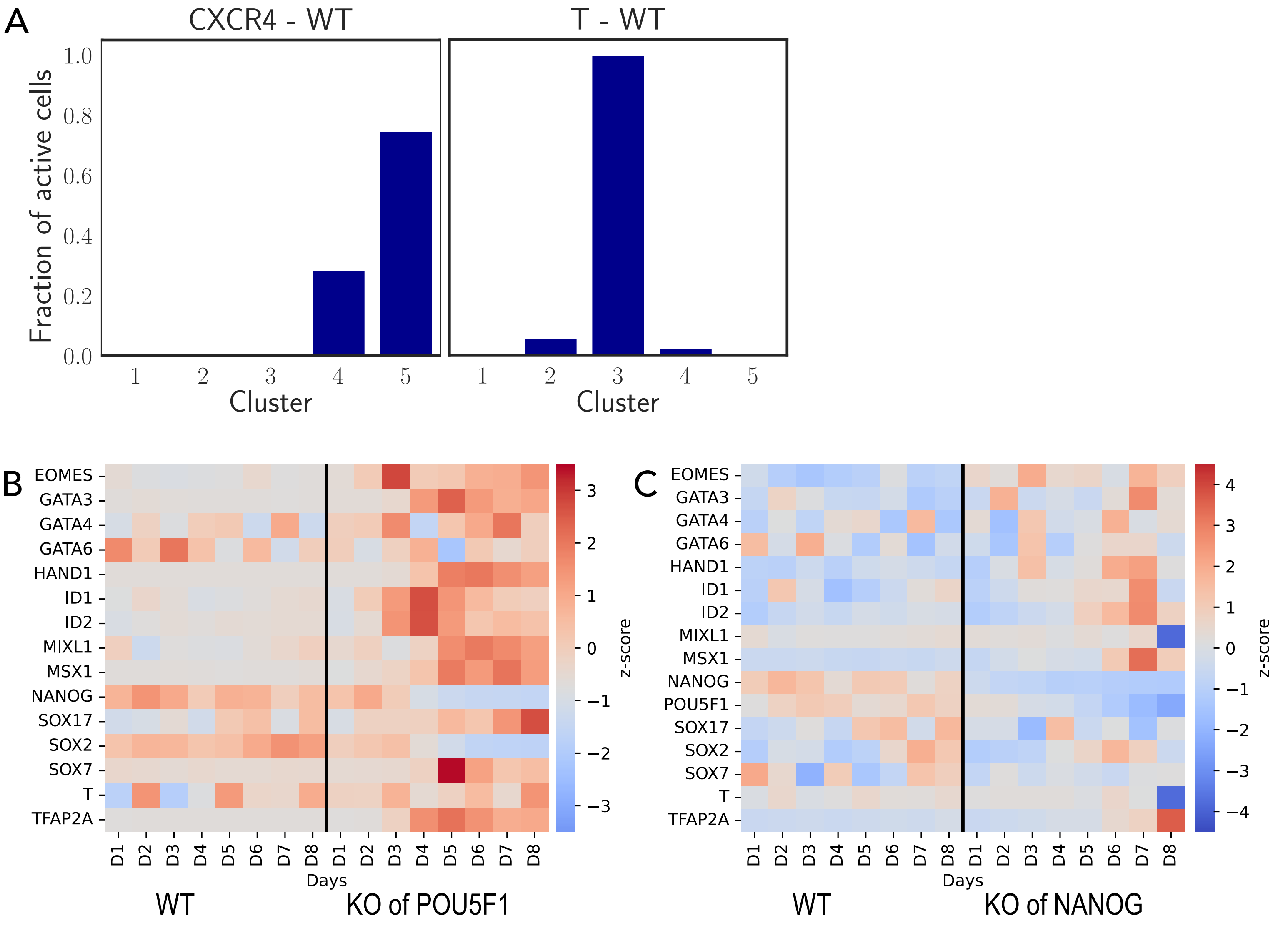
A
CXCR4 - WT
T - WT
Fraction of active cells
1.0
0.8
0.6
0.4
0.2
0.0
1
2
3
4
5
Cluster
Cluster
B
EOMES
GATA3
GATA4
GATA6
HAND1
ID1
ID2
MIXL1
MSX1
NANOG
SOX17
SOX2
SOX7
T
TFAP2A
D1 D2 D3 D4 D5 D6 D7 D8 D1 D2 D3 D4 D5 D6 D7 D8
Days
WT
KO of POU5F1
z-score
C
EOMES
GATA3
GATA4
GATA6
HAND1
ID1
ID2
MIXL1
MSX1
NANOG
POU5F1
SOX17
SOX2
SOX7
T
TFAP2A
D1 D2 D3 D4 D5 D6 D7 D8 D1 D2 D3 D4 D5 D6 D7 D8
Days
WT
KO of NANOG
z-score

S7 Figure. Validation of IGNITE predictions against independent human knockout datasets.

A. Fraction of cells with active CXCR4 (left) and T (right) in each cluster from GA data generated by IGNITE.

B. Standardized expression levels (z-scores) of selected genes across differentiation time points in WT (left) and POU5F1 KO (right) conditions. Data from Wang et al. [1].

C. Standardized expression levels (z-scores) of selected genes across differentiation time points in WT (left) and NANOG KO (right) conditions. Data from Wang et al. [1].

References

[1] Zheng Wang et al. "Distinct Lineage Specification Roles for NANOG, OCT4, and SOX2 in Human Embryonic Stem Cells". In: *Cell Stem Cell* 10.4 (Apr. 2012), pp. 440–454. ISSN: 1934-5909. DOI: 10.1016/j.stem.2012.02.016. URL: https://doi.org/10.1016/j.stem.2012.02.016.
