# Supplementary material for: Unveiling gene perturbation effects through gene regulatory networks inference from single-cell transcriptomic data
Source: PLoS Comput Biol. 2026 Apr 15;22(4):e1014067. doi: 10.1371/journal.pcbi.1014067 (PMC13082667; doi:10.1371/journal.pcbi.1014067)
Supplement: S1 Table — (PDF) [file pcbi.1014067.s008.pdf]

S1 Table

Benchmark task	mPSC dataset	hPSC dataset	Reference
GRN inference and WT data generation	CMD minimization; Spearman correlation between input and generated gene activity; agreement of cluster composition	CMD minimization; reproduction of WT gene activity structure in PCA space; preservation of cluster fractions	Fig. 2A–G; Fig. 7A–D
GRN validation against known interactions	Recovery of experimentally validated interactions; Fraction of Correctly Inferred interactions (FCI); comparison with other inference methods	Recovery of known regulatory interactions (TRRUST); consistency with established endoderm differentiation logic	Fig. 4A–C; Table 3; Fig. S6C–D
WT generative capacity	Preservation of naïve and formative cell fractions; overlap between input and generated data in PCA space	Preservation of differentiation trajectory structure; correct fraction of cells in the clusters	Fig. 2E–G; Fig. 7C–D
KO perturbation prediction	Gene-level KO–WT differences; Spearman and Fraction of Agreement (FoA) correlation with experimental $\log_2$FC	Cluster-level shifts under KO conditions; Differentiation Score (DS) comparison with experimental perturbations	Fig. 3A–E; Tables 2–3; Fig. 8A–C

Table S1: Side-by-side summary of the benchmark tasks and validation strategies applied to the mouse (mPSC) and human (hPSC) datasets. While dataset-specific metrics are used, the same benchmarking framework is applied across species and experimental platforms.
