# Supplementary material for: Unveiling gene perturbation effects through gene regulatory networks inference from single-cell transcriptomic data
Source: PLoS Comput Biol. 2026 Apr 15;22(4):e1014067. doi: 10.1371/journal.pcbi.1014067 (PMC13082667; doi:10.1371/journal.pcbi.1014067)
Supplement: Attachment — Submitted filename: 148168_1_rebuttal_3553035_t4h3ks.pdf [file pcbi.1014067.s009.pdf]

Revision Plan

Review COMMONS

Manuscript number: RC-2024-02557
Corresponding author(s): Graziano, Martello

1. General Statements [optional]

This section is optional. Insert here any general statements you wish to make about the goal of the study or about the reviews.

We appreciate the reviewers' valuable feedback and thank the Review Commons editor for handling our manuscript. We thank the reviewers for their comments which have been extremely helpful, and we are pleased that the reviewers understood that we are proposing a novel "*single framework capable of inferring, simulating and perturbing gene expression*" using single-cell RNA-seq (scRNA-seq) data as the only input. As scRNA-seq is becoming a standard technique for transcriptional analysis of cell populations, IGNITE can be applied to several different biological systems. Thus, IGNITE will be of interest to "*a broad community of computational biologists and biology labs interested in gene regulation using scRNA-seq data*".

Three important issues were raised by more than one reviewer, and we have addressed them. The first issue concerns the necessity of using additional gene expression datasets to test the robustness of IGNITE performance across different biological systems and scRNA-seq techniques. To address this, we have applied IGNITE to a dataset [1] previously analysed with SCODE (the current state-of-the-art method), as suggested by Reviewer 1.
The second point involves understanding how IGNITE performance scales with an increasing number of genes. We have already investigated this aspect, finding that the computational time increases quadratically with the number of genes considered, as observed with data points for 24, 50, 94, and 200 genes. Importantly, even large GRNs (e.g., 200 genes) could be analysed on a standard laptop (MacBook Pro with an Apple M1 Pro processor and 16 GB of RAM).
The last point concerns the availability of the code and data generated in this study. We have made the code available on GitHub (https://github.com/CleliaCorridori/IGNITE) and the scRNA-seq data have been already deposited and will be made available to reviewers and the community upon publication.
In the subsequent sections, we provide detailed responses to each point raised by the reviewers.

[1] Chu LF, Leng N, Zhang J, Hou Z, Mamott D, Vereide DT, Choi J, Kendziorski C, Stewart R, Thomson JA, et al. Single-cell RNA-seq reveals novel regulators of human embryonic stem cell differentiation to definitive endoderm. Genome Biol. 2016;17:173. doi:10.1186/s13059-016-1033-x

Revision Plan

2. Description of the planned revisions

Insert here a point-by-point reply that explains what revisions, additional experimentations and analyses are planned to address the points raised by the referees.

We have carefully evaluated all reviewers' comments and implemented the necessary revisions to address the key points raised.

3. Description of the revisions that have already been incorporated in the transferred manuscript

Please insert a point-by-point reply describing the revisions that were already carried out and included in the transferred manuscript. If no revisions have been carried out yet, please leave this section empty.

Reviewer #1 (Evidence, reproducibility and clarity (Required)):
Summary

Corridori and colleagues propose IGNITE, a novel method to recover Gene Regulatory Networks (GRN) from single cell RNA-sequencing (scRNA-seq) data. Their method solves the inverse Ising problem generating a cohort of candidate GRN optimising it to minimise the difference to the input expression matrix. Authors report the IGNITE is able to predict wild type data and simulate both single and multiple gene knockouts. Authors benchmark this method on a in-house data set of differentiating pluripotent stem cells (PSC). They focus on a small set of genes known to be involved in PSC differentiation into formative cells. Authors benchmark IGNITE against state of the art tools (SCODE, MaxEnt and CELLORACLE). They evaluate IGNITE ability to predict wild type gene expression by comparing their data with experimental data and with SCODE. They conclude the tool has generative capacity comparable with SCODE. They also evaluate IGNITE ability to recover known interactions with respect to other tools without finding it to significantly outperform them.

Major comments
- Are the key conclusions convincing?

Conclusions appear convincing although model generalizability could be shown in a more thorough manner. For instance, analysing some other publicly available dataset could help demonstrate hyperparameters effects on GRN predictions and their robustness across different experiments.

We thank the reviewer for finding our conclusions convincing. We recognise the importance of demonstrating the robustness and effectiveness of our approach across different datasets. To address this, as they suggested, we benchmarked our method using a completely independent

dataset. Specifically, we analysed human PSC differentiation towards endoderm, profiled with a different scRNA-seq platform (Chu et al, doi: https://doi.org/10.1186/s13059-016-1033-x). From this dataset, we inferred a GRN composed of 98 transcription factors selected unbiasedly as the most variable regulators.

The inferred GRN successfully simulated wild-type data and accurately predicted the effects of gene knockouts. Notably, KO predictions were consistent with experimental observations reported in two independent studies (see **section 3.9** and **new Fig. 8**).

These novel validations further support IGNITE's generalizability performance and provide insights into the role of hyperparameter choice under different experimental conditions, which will help guide future users in applying IGNITE to diverse datasets and biological questions.

- Should the authors qualify some of their claims as preliminary or speculative, or remove them altogether?

Claims are well supported by data.

We are glad that the reviewer appreciated our efforts.

Would additional experiments be essential to support the claims of the paper? Request additional experiments only where necessary for the paper as it is, and do not ask authors to open new lines of experimentation.

I think the work would benefit from an additional benchmark on a different cellular system. This experiment would show how hyperparameters generalise across datasets and would provide potential users insights how to tweak them.

As anticipated above, we have included an additional benchmark using a dataset [1] previously employed by Matsumoto et al. to evaluate SCODE's performance, human PSC differentiation toward endoderm (see **new Fig. 6-8** and **S5-S7**).

Also, how does the model scale with the number of genes? A benchmark on computation time and resources required to infer GRN of growing size would be valuable in the adoption of this tool.

Thank you for your pertinent question regarding the scalability of our model. We generated GRNs of increasing size from 24 to 200 genes, using as input our large scRNA-seq data (~9500 cells). We have performed this analysis and measured the computation time (see **Fig. R1**) on a standard laptop computer (MacBook Pro with an Apple M1 Pro processor and 16GB of RAM). The computational time fits a quadratic trend as the number of genes considered increases, reaching ~12 hours for the largest GRN, which is an acceptable computational time (see **Section 3.6**, "Evaluating Prior Knowledge Integration and Scaling properties").

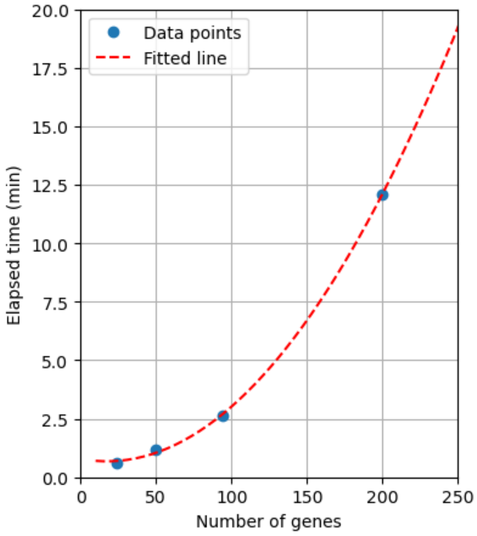

Figure R1: Elapsed computational time required by IGNITE to infer GRNs of increasing size, measured on a standard laptop computer (MacBook Pro with an Apple M1 Pro processor and 16 GB of RAM). Blue dots represent measured values, and the red dashed line is the quadratic fit (R2=0.9997)

In addition, I think the GRN comparison benchmark presented in section (3.4) would benefit from a quantitative discussion. Authors show inferred GRNs in Figure 4 and S5. For instance, measuring matrix similarity (when appropriate) would help understanding how predicted GRN compare. I understand authors attempt to do so by focusing on validated interactions and computing the fraction of correctly inferred interactions (FCI) but I think a measurement of the overall similarity (eg. Pearson correlation) would add on this.

We appreciate your input on the potential benefit of incorporating matrix similarity measures to enhance the understanding of how predicted GRNs compare. In line with this, we computed GRNs similarity using Spearman correlation, comparing each method against IGNITE. As reported in **Tab. 4**, MaxEnt and CellOracle exhibit moderate positive correlations with IGNITE, whereas SCODE shows no significant correlation.

Another comment regards the dependency between Correlation Matrices Distance (CMD) and FCI, shown in Figure 5. I understand that IGNITE GRN that maximises FCI are not the same that minimise CMD. However, it looks like GRN that maximise FCI have higher value in terms of biological information. I wonder whether optimization for one or the other metric could be left to the end user as a tunable parameter.

We agree that allowing users to choose or tune the metric based on their needs would be beneficial when prior knowledge is available. This is already implemented in the code, as shown for the GRN selection based on FCI.

In the revised manuscript, we replaced FCI with Spearman correlation between simulated and experimental KO–WT differences as a more informative and quantitative measure of prediction accuracy (see **Fig. 3E** and **Tab. 5**). Notably, GRNs selected by CMD achieved robust and statistically significant correlations across KO conditions, whereas FCI-selected GRNs displayed lower and more variable performance. This demonstrates that CMD provides a reliable optimisation criterion, yielding consistent KO predictions without requiring prior knowledge of validated interactions.

Authors should discuss why the expression of some genes does not follow the expected trends (Fig 1C vs Fig S1A). Out of the 24 genes they select for their analysis, at least four do not follow the expected trends: Sox2, according to literature, is a Naive gene, however, in Figure 1C its gene expression pattern is more similar to Formative late genes. Other genes with similar "unexpected" patterns are Zic3, Etv4 and Sall4.

We thank the reviewer for this important observation. We agree that certain genes, such as Sox2, Zic3, Etv4, and Sall4, exhibit gene expression patterns that appear discordant between the z-score of bulk RNA-seq and the z-score of the single-cell RNA-seq dataset (**Fig. 1C**). However, the two datasets, although both representing mouse PSC differentiation, originate from independent experiments and employ distinct cell lines and protocols; some degree of inter-dataset variability is therefore expected (see **Fig. R2**). Despite these differences, the correlation matrix highlights consistent temporal patterns between the two datasets.

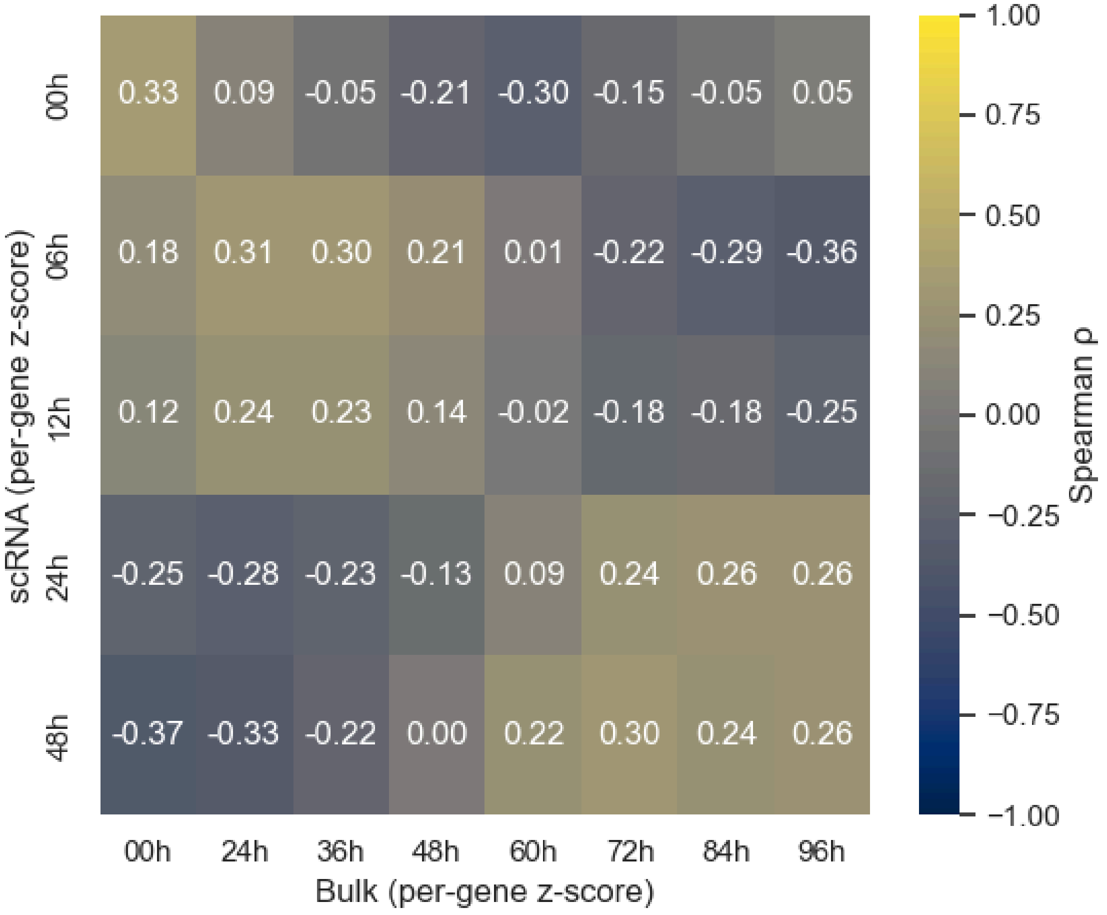

***Figure R2**: Spearman correlation between bulk and scRNA-seq gene expression dynamics across time points. Correlation coefficients are computed between each averaged scRNA-seq time point (rows) and each bulk RNA-seq time point (columns), using per-gene z-scored expression values for the 24 selected signature genes (normalized as log(CPM+1) for bulk and log-normalized for single-cell). Z-scoring was performed independently in each dataset to highlight relative temporal patterns rather than absolute expression levels.*

In the case of Sox2, its sustained or even increased expression during early differentiation toward the neuroectoderm lineage has been previously reported and is biologically consistent. Our single-cell dataset captures early lineage priming events that may not be equally resolved in the bulk data, explaining the negative correlation observed for Sox2 (Spearman ρ = –0.8). However, in both datasets Sox2 does not display the typical decay pattern observed for naïve pluripotency markers such as Nanog and Klf2 (**Fig. R3**). While these genes show a marked and consistent downregulation over time, Sox2 levels remain relatively sustained. This suggests that Sox2 does not behave as a canonical naïve marker in this context, which motivated its reclassification into the “Other” category in our figures and analyses.

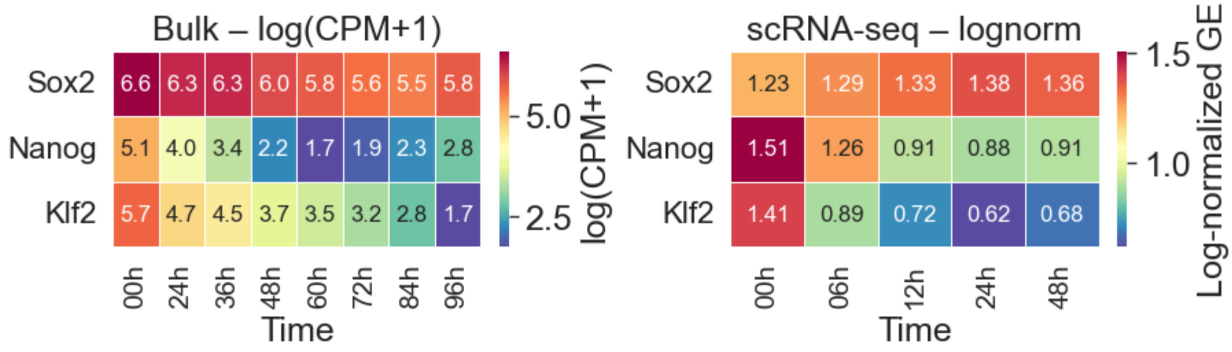

***Figure R3**: Gene expression (log(CPM+1) for bulk and log-normalized single-cell) for the four specific genes in the initial time points of the two datasets.*

While gene-specific discrepancies in expression dynamics exist, they can be explained by biological and technical variability between systems and differences in temporal resolution. Since a detailed bulk–single-cell comparison was out of the scope of the manuscript, we have removed from the revised version the comparison with the bulk dataset (**previous Fig S1A**). However, these do not affect the overall consistency of the temporal trends observed across datasets.

Are the suggested experiments realistic in terms of time and resources? It would help if you could add an estimated cost and time investment for substantial experiments.

I think suggested experiments are doable as long as authors get publicly available data, i.e. the in-house dataset they generated for this study is enough to show applicability. For example datasets analysed in SCODE paper (https://doi.org/10.1093/bioinformatics/btx194) could be used as second benchmark. The point of applying the tool to another dataset is to show how it generalises across different biological systems, experiments and, potentially, sequencing technologies.

Testing our approach on the dataset suggested by the reviewer has been a substantial undertaking. However, we thought it would have been very important to test the generalizability of our approach beyond the dataset we generated and analysed in the submitted version of our manuscript.

- Are the data and the methods presented in such a way that they can be reproduced?

The methods section is really clear. To enable reproducibility both raw scRNA-seq data, the IGNITE source code and code written to benchmark it should be released in the public domain in appropriate repositories (eg. ENA, GitHub, Binder etc).

We have made the IGNITE source code available on GitHub at https://github.com/CleliaCorridori/IGNITE, along with the code used for SCODE and CellOracle

applied to our data. In accordance with standard practice, the raw scRNA-seq has been already deposited in appropriate repositories and will be accessible upon acceptance of the manuscript.

- Are the experiments adequately replicated and statistical analysis adequate?

Yes.

Minor comments

- Specific experimental issues that are easily addressable.

Related to the Sox2 expression pattern is the binarization shown in Figure 2D. How is it possible that Sox2 is always marked as active? Could the authors clarify how these outlier behaviours emerge and propose mitigation strategies, if any?

Sox2 is expressed throughout the entire time course, with a slight increase around 24h. This is why in the binarization it is always active.
This behavior is not unexpected biologically, as Sox2 remains active during early transitions, particularly in cells primed for neuroectodermal commitment (as discussed in our response above regarding Sox2 dynamics across datasets).

In section 5.11.2 it is unclear if xi are in log scale or not. Since the model starts from binarised, log transformed expression values, should not generated ones be in the same scale as the input?

The reviewer is correct, the generated values are in the same scale as the input. So it is gene activity (binarised gene expression) for IGNITE and gene expression for SCODE and CellOracle.
We edited **Sec. 5.10.2** (Sec. 5.11.2 in the first version of the manuscript) and indicated more clearly what x_i indicated (see *lines 958-963*).

- Are prior studies referenced appropriately?

Yes, referencing is clear.

- Are the text and figures clear and accurate?

Yes, figures appear to be clear, readable and well documented both in captions and main text.

- Do you have suggestions that would help the authors improve the presentation of their data and conclusions?

Section 3.3 could be improved by better describing experimental datasets. Only in the methods section it is clearly stated that experimental data for single KO experiments were retrieved from the literature.

We thank the reviewer for this suggestion. In the revised manuscript, we have clarified and expanded Section 3.3, now **Sec. 3.4**, to make the provenance and processing of the experimental KO data explicit within the Results, not only in Methods.

Check typesetting:
- parenthesis missing in Eq. 1
- Leftover $ in section 3.1
- Parenthesis missing in Section 3.3
- Misplaced comma in section 5.2.1

Thank you for your detailed review and for pointing out the typesetting issues. We have addressed all the typographical errors that you highlighted, except for the missed parenthesis. After a thorough review, we believe that the parenthesis in Eq. 1 is correctly included. However, if you have a specific concern or location in mind where the parenthesis might be missing, please let us know so that we can re-evaluate it.

Reviewer #1 (Significance (Required)):

Significance
- Describe the nature and significance of the advance (e.g. conceptual, technical, clinical) for the field.

The paper presents a method to infer GRN from scRNA-seq data alone. Applications include GRN prediction and their perturbations. This paper represents a technical advance in the field as it is the first application of the inverse Ising problem GRN inference.

- Place the work in the context of the existing literature (provide references, where appropriate).

The paper itself presents the landscape of GRN inference tools using scRNA-seq data: SCODE, MaxEnt and CELLORACLE. More tools exist, for instance SCENIC (https://doi.org/10.1038/nmeth.4463) mainly relies on co-expression matrices. Other tools exist but require additional data types e.g. GRaNIE and GRaNPA (https://doi.org/10.15252/msb.202311627) leverage on physical interaction data (ATAC-seq, ChIP-seq). Similarly DeepFlyBrain uses deep neural networks to infer eGRN in Drosophila (https://doi.org/10.1038/s41586-021-04262-z). The value of tools like IGNITE and its competitors is that they do not require additional data types, which, in turn, helps in controlling experimental costs.

- State what audience might be interested in and influenced by the reported findings.

The paper might be of interest to biologists interested in regulation of gene expression. The tool might turn out to be useful in planning experimental work by guiding the choice of perturbations to introduce in experimental systems.

- Define your field of expertise with a few keywords to help the authors contextualise your point of view. Indicate if there are any parts of the paper that you do not have sufficient expertise to evaluate.

I am a computational biologist.

I have no sufficient expertise to evaluate the mathematical details of the method.

Reviewer #2 (Evidence, reproducibility and clarity (Required)):

Corridori et al introduce IGNITE, a computational framework to infer gene regulatory networks (GRNs) from scRNA-seq data leveraging the kinetic Ising model, which can be used to simulate synthetic gene expression and perform in-silico knockout experiments. Other similar frameworks exist, but none combine these three aspects together. The authors have generated a scRNA-seq of murine ESCs differentiation which they use to compare their method with others. Specifically they show that they can infer known regulatory interactions, that they can generate similar data than the original and that it can potentially predict gene expression changes in transcription factor knock-out perturbations.

Major comments:

-Many of the authors' claims are backed by qualitative results and not properly quantified. In Fig2, authors qualitatively compare intra gene correlations between genes for the original data and their prediction. Instead of just visualising they should compute and report the Spearman correlation between the original expression and the predicted one.

We thank the reviewer for this helpful suggestion. First, we apologize for the confusion: in fact, we did perform a quantitative comparison of intra-gene correlations between the original and predicted data by using the Correlation Matrices Distance (CMD), which is based on the Euclidean distance between correlation matrices. We also carried out statistical validation of CMD values (*lines 170-177*). We acknowledge that the statement "generated by the IGNITE-selected GRN and found them qualitatively congruent in terms of correlation" in the first version of the manuscript could be confusing, so we edited it in the manuscript (*lines 178-185*). Second, following the reviewer's recommendation, we have now added an additional measure of similarity. It is the Spearman correlation between the average original expression values and the average predicted ones. The correlation coefficient for the mean gene activity values is 0.99

(p-value < 2.2 × 10^{-16}), confirming a very high inter-gene correlation between input and generated data (*lines 178-185*).

The Fraction of Agreement is not a good metric to compare knockout predictions since it is completely dependent on the class imbalance of signs, for example if the selected genes are 75% positive and 25% negative, a naive predictor that only outputs positive predictions will still have a high score. Instead, the authors should quantify this with Spearman correlation or RMSE and compare across methods.

We thank the reviewer for this comment. We agree that the Fraction of Agreement (FoA) is sensitive to class imbalance and may not be the most appropriate metric. Following the suggestion, we have now evaluated the model performance using Spearman correlation, which captures the monotonic relationship between predicted and experimental data and is more robust to class imbalance.
The results obtained from Spearman correlation are consistent with those derived from the FoA, reinforcing the robustness of our model despite the noted limitations of the FoA. These findings are detailed in the revised manuscript (see **Tab. 2**).

In FigS4a-b the authors qualitatively claim that other methods could not predict the expected cell composition, which they should quantify and report the values across methods.

We thank the reviewer for this constructive suggestion. We agree on the importance of providing a quantitative assessment of cell composition predictions. To address this, in the revised manuscript we quantified the fraction of naïve and formative cells predicted by SCODE and CellOracle following the triple knockout (*lines 257-264*). SCODE generated KO data with only a slight increase in naïve cells compared to WT (from 21% in WT to 28% in KO condition), whereas CellOracle produced a moderate shift (from 18% in WT to 48% in KO condition) but still underestimated the naïve fraction relative to experimental data, where most cells remain naïve. By contrast, IGNITE simulations reproduced the expected composition, with naïve cells reaching 79%. To present these results more clearly, we replaced the previous Fig. S4 with new supplementary heatmaps (**Fig. S2F and H**), which illustrate the cell state distributions underlying these percentages. This addition directly addresses the reviewer's concern and strengthens the quantitative comparison across methods.

When comparing against the ground truth network, the fraction of correctly inferred interactions is technically the same as precision but is ignoring recall. I suggest the authors compute precision, recall and a combined F1 score to compare the evaluated methods.

We thank the reviewer for this thoughtful comment. The ground truth network we used is based on the combination of different experimental data. We first used ChIP-seq, which detects the presence of protein X on the promoter of a target gene Y. Then we used gene expression data (e.g. RNA-seq) generated upon inactivation of protein X and asked whether the expression of

gene Y was affected. By combining the two types of data we obtained a list of “true” interactions, those in which X directly regulates the expression of Y.
However, both techniques are prone to false negatives. So we might have failed to detect an interaction, but it does not mean that such interaction does not exist. It is more challenging to establish the absence of a given interaction.
Computing recall and F1 scores would require a comprehensive list of both present and absent interactions, which is currently not available. For this reason, we employed the Fraction of Correctly Inferred Interactions (FCI), which evaluates how well methods recover the known interactions, including their regulatory sign (activation or repression).

Authors claim that the method is scalable to a larger number of genes but no data is provided, they should show how their method compares to others when using a different number of cells and number of genes at memory usage and running time.

We acknowledge the need to test the scalability of IGNITE with varying numbers of genes and cells. To this end, we generated GRNs of increasing size from 24 to 200 genes, using as input our large scRNA-seq data (~9500 cells). We have performed this analysis and measured the computation time on a standard laptop computer (MacBook Pro with an Apple M1 Pro processor and 16GB of RAM).
The computational time fits a quadratic trend as the number of genes considered increases, reaching ~12 hours for the largest GRN, which is an acceptable computational time (See **Section 3.6**, “Evaluating Prior Knowledge Integration and Scaling properties”).
To assess scalability with respect to cell number, we randomly subsampled 500 cells (~5% of the dataset) and re-inferred GRNs. As shown in **Tab. 6** (See **Section 3.6**), KO prediction performance (Spearman correlations) remained comparable to that obtained using the full dataset, demonstrating that IGNITE preserves predictive accuracy on KO simulations even when applied to limited numbers of cells. While informative, a systematic benchmarking of scalability across other inference methods was beyond the scope of this study.

-The authors need to better describe which tests were performed when talking about significance, which thresholds and which corrections, if any, were employed.

We thank the reviewer for raising this important point. We agree that it is essential to clearly specify which statistical tests were used, including thresholds and corrections, when reporting significance.

We would like to stress that in some parts of the manuscript such information was already present in the previous version. For example, for the analysis relative to **Figure 2A**, we had already reported that the difference between CMD values of IGNITE and null models was assessed with a two-sample *t-test* (*lines 175-177*).

In response to the reviewer’s suggestion, we have now further clarified the statistical procedures in other parts of the manuscript. For example, for the Fraction of Agreement (FoA, **Fig. 3** and

Tab. 3) we now explicitly state that statistical significance was assessed with a **binomial test** against a random null model.

Moreover, the current version of the manuscript includes several new analyses, for which we now provide full statistical validation. These include, for example, the Spearman correlation between input and generated data (**Fig. 2B–C**), Spearman correlation between predicted and experimental KO–WT differences (**Fig. 3 and Tab. 2**), as well as all analyses on the human PSC dataset (**Figs. 6–8**), where we report CMD vs null comparisons (two-sample *t*-test), KO–WT correlations (Spearman test), and differentiation score comparisons.

We would like to point out that the statistical tests used throughout the manuscript are described in detail in the *Methods* section (e.g., permutation test for network significance, binomial test for FoA, Spearman rank correlation test for KO–WT predictions, two-sample *t*-test for CMD comparisons). In the *Results* section, whenever we refer to “significant differences” or “statistical significance,” these statements directly rely on the corresponding tests described in *Methods*.

-To reduce the number of dimensions of scRNA-seq data the authors use t-SNE and then from the obtained result UMAP to project the data into a lower dimensional space. This is fundamentally wrong since distances are not well preserved in t-SNE. Instead the authors should first employ PCA and then UMAP. Additionally, the authors use UMAP distances in the Slingshot pseudotime calculation. Similar to t-SNE, UMAP distances have no real meaning and should only be used for visualization purposes. Instead, the authors should provide Slingshot the obtained PCA embeddings.

We thank the reviewer for the thoughtful suggestion. It is true that the different dimensionality reduction methods may affect the results obtained with Slingshot. We are aware of the fact that each method captures only part of the experimental data.
While PCA aims to preserve as much variance as possible, it does so by sacrificing the less significant components, which may contain crucial distance information. This distortion occurs because PCA emphasises global structure by maximising variance, but may not maintain the original pairwise distances between data points. This is particularly evident when the data has complex structures or when the intrinsic dimensionality is much higher than the reduced dimensions. The more dimensions you reduce, the greater the potential for distortion. Although t-SNE and UMAP prioritise local over global structures, they are particularly effective for clustering tasks and can separate distinct cell types well, as highlighted in recent evaluations (https://www.frontiersin.org/journals/genetics/articles/10.3389/fgene.2021.646936/full).
As t-SNE and UMAP conserve the local distance among cells, we believe it is a fair choice for our dataset, but PCA might be preferable for other datasets with multiple separate cell populations. Indeed, our mouse dataset cells were sampled frequently along a short linear transition, without bifurcations. Cells appear as a single “cloud” ordered according to the real temporal progression, thus we would tend to exclude that t-SNE and UMAP caused a major distortion in the dataset. We did not use PCA before UMAP because for this dataset PCA was capturing a small fraction of the variance (see **Fig. R4**), and failed to clearly separate cells from

different time points. In contrast, t-SNE successfully captured the temporal structure in the data, leading us to select it as the more appropriate choice for this specific case (see **Fig. R5**).

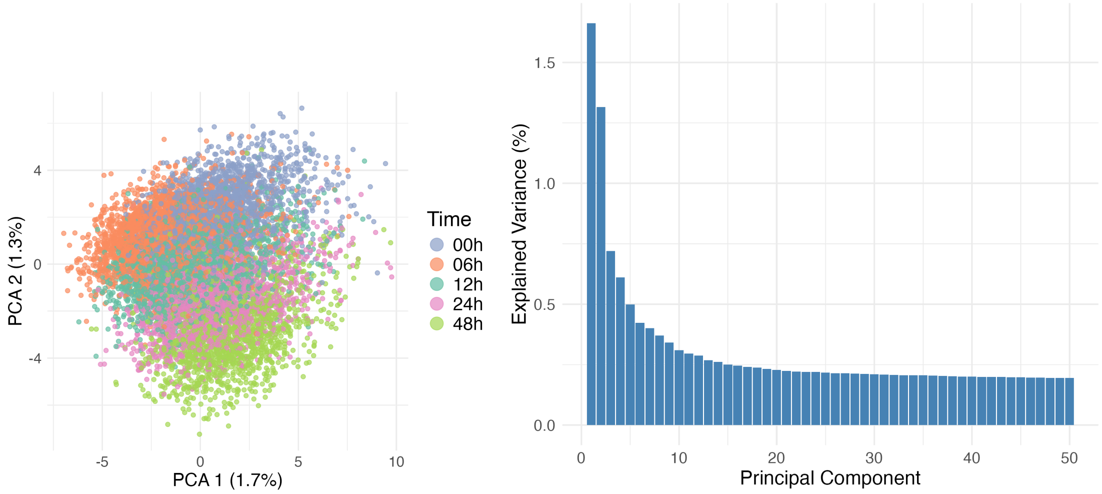

Figure R4: ***a)*** *two-dimensional PCA of the input experimental dataset (scRNA-seq data with Log-Norm). Each point represents an individual cell colored according to its sampling time. PCA1 and PCA2 indicate the two dimensions of the PCA space.* ***b)*** *Variance explained by the first 50 principal components from log-normalized gene expression data.*

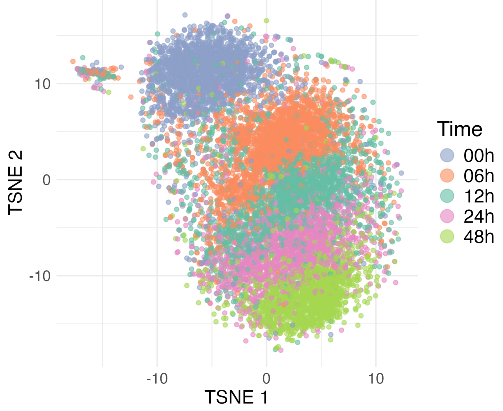

Figure R5: *Two-dimensional t-SNE of the input experimental dataset (scRNA-seq data with Log-Norm). Each point represents an individual cell colored according to its sampling time. t-SNE1 and t-SNE2 indicate the two dimensions of the t-SNE space.*

Nevertheless, in our new analysis of a second dataset—human PSCs differentiating towards definitive endoderm (doi: https://doi.org/10.1186/s13059-016-1033-x)—we adopted a different strategy and used PCA embeddings as input for Slingshot, in line with the reviewer's recommendation. In this case, PCA was able to capture the underlying temporal structure of the data (see **Fig. S5A**).

Finally, we would like to clarify that pseudotime values in our pipeline are used only to identify locally similar cells for the construction of Mini-Bulk profiles. We are not interested in the actual trajectories and position of cells in space.

-Dictys (PMID: 37537351) is a known GRN inference method that also can simulate gene expression but is missing in the benchmark, the authors should add it to the method comparison.

Dictys is a method requiring multi-omics data as input. At least, scRNA-seq and single-cell ATAC-seq are needed. Our experimental data from PSCs could not be analysed with Dictys, as we do not have matching single-cell ATAC-seq data.
Furthermore, as acknowledged by all reviewers, a strength of IGNITE is that it requires only scRNA-seq as input. Thus, a comparison with Dictys would be not informative.
Assuming that Dictys performs better when single-cell multi-omics data are available, IGNITE would still be the preferred choice for most biological systems, for which only scRNA-seq is available.

-The current manuscript is not reproducible since it is missing the method's code, the code to reproduce the figures and the generated scRNA-seq data.

We have made the IGNITE source code available on GitHub at https://github.com/CleliaCorridori/IGNITE, along with the code used for SCODE and CellOracle applied to our data. The IGNITE code can be used to generate the figures and the data.

-Authors claim that the method is scalable to a larger number of genes but no data is provided to back this claim. They should show how their method compares to others when using a different number of cells and number of genes.

This point duplicates the scalability comment above, and we already addressed it.

Minor points:

-In the introduction, authors mention multimodal GRN inference methods but do not provide any references.

We thank the reviewer for the observation. We have now added the relevant references to multimodal GRN inference methods in the Introduction (*lines 19-20*).

-In Table 1, CellOracle is annotated as not being able to do multiple KO which is wrong.

We apologize for any confusion caused by the table. You are correct that CellOracle does support multiple knockouts, even though this capability was not explicitly analysed in the paper

(https://doi.org/10.1038/s41586-022-05688-9). We have clarified this in the revised manuscript by updating the caption (see **Tab. 1**).

- Additionally, the authors mention that IGNITE uses no prior knowledge which is not really true since it requires pseudotime ordering. The authors should add a column to Table 1 whether methods require pseudotime.

Thank you for your helpful suggestion. We have added a column in Tab. 1 indicating whether each method requires pseudotime information, as recommended.
However, we point out that the pseudotime information in IGNITE is computed from single-cell RNA-seq data using an algorithm with no need for external prior knowledge. In our context, prior knowledge refers to the integration of additional information (e.g., ATAC-seq data or the effects of KOs) beyond the single-cell data.

-It is unclear what the dashed arrow of Fig1b means.

We thank the reviewer for pointing this out. We have revised **Fig. 1B** to enhance clarity.

Moreover, plotting gene expression values on top of UMAPs can be misleading, instead authors should plot the gene expression distributions binned by pseudotime.

We thank the reviewer for this helpful suggestion. In the revised manuscript we have now included plots of gene expression in pseudotime, which more clearly illustrate expression changes along the differentiation trajectory. Specifically, for the mouse dataset this is now shown in **Fig. 1F**, and for the newly added human dataset in **Fig. 6E**.

-The authors report a p-value of 1.04x10-171 which is below detection limit (see PMID: 30921532). Authors should change it to an interval such as p < 2.2×10-16.

Thank you for pointing this out. We appreciate the suggestion and updated the text to use this p-value interval when necessary, as recommended (e.g. in *line 165*).

-To make CellOracle results easier to interpret and more comparable, authors should run it at the atlas level instead of at the cell type level, this way generating only one GRN. This can be achieved by assigning the same cluster label to all cells.

The authors of CellOracle recommend generating a distinct GRN for each cell cluster. Predictions are then generated exploiting all GRNs. This should be a strength of their method. For this reason, we think that using the method as recommended by the authors is the fairest way to compare it against IGNITE.
There are several other parameters that differ between CellOracle and IGNITE (e.g. using imputation to deal with zero inflation rather than calculating a Mini-Bulk) and testing all possible combinations of parameters is beyond the scope of our current study.

-Experimental values in FigS3b seem to have been repeated and do not match the previous ones for IGNITE and SCODE.

Thank you for pointing this out. We have corrected the typo (**Fig. 3 A and B**) and updated the figure and the corresponding FoA results for CellOracle (**Tab. 3**). The revised version has been included.

-It is unclear what the different circles mean in Fig5b.

We thank the reviewer for pointing this out. In the revised version we simplified the figure by removing the individual data points and retaining only the boxplots. This eliminates any possible confusion about symbol meaning while still clearly representing the distribution of values

Reviewer #2 (Significance (Required)):

This manuscript is an incremental and methodological work for specialised audiences. Its strengths are that the authors employ kinetic Ising model for GRN inference and that they provide a single framework capable of inferring, simulating and perturbing gene expression. The main limitations are that the claims should be better quantified and that the code and data need to be made accessible.

Reviewer #3 (Evidence, reproducibility and clarity (Required)):

SUMMARY

The manuscript presents IGNITE (Inference of Gene Networks using Inverse kinetic Theory and Experiments), an unsupervised machine learning framework for constructing gene regulatory networks from single-cell RNA sequencing (scRNA-seq) data. IGNITE utilises a kinetic inverse Ising model to infer gene interactions from binarised expression data and can predict genetic perturbation effects, such as those from knockout experiments. Although the application of inverse Ising models to network reconstruction is not entirely novel, IGNITE's specific implementation and its application to single-cell RNA sequencing data represent a new development. The method is tested on the transition from naive to formative states in murine pluripotent stem cells, a system the authors are highly knowledgeable about, and its performance is compared to state-of-the-art alternative methods.

MAJOR CONCERNS

My concern regards the generality of the method, particularly the entire pipeline presented, and the fairness of the performance comparison. These concerns can be easily addressed by the

authors by better explaining their choices and their general applicability, and by toning down the conclusions about the comparison with existing inference methods.

Thank you for your thoughtful comments. To address your concerns about the generality of the method and the fairness of the performance comparison, we have extended our analysis to include an additional dataset: the differentiation of human PSCs toward definitive endoderm [1]. This dataset, which was also used by Matsumoto et al. to assess SCODE's performance, represents a distinct biological system and was generated using a different platform (Fluidigm C1) and a significantly lower number of cells (~500) compared to our mouse dataset.

This additional benchmark allowed us to evaluate IGNITE's robustness across experimental settings, demonstrating its applicability to datasets with varying cell numbers, biological contexts, and technical platforms. Moreover, in this analysis, gene selection for GRN inference was performed in an unbiased manner—unlike the mouse dataset, where gene selection was guided by prior literature—further supporting the general applicability of our approach.

We have also revised the manuscript to provide a clearer description of the comparison methods and parameters used, and we have moderated our conclusions to reflect a more balanced assessment of IGNITE's performance relative to existing inference methods.

The pre-processing steps are extensive, and their rationale is not always clear, though the results heavily depend on this analysis. Several steps appear to involve arbitrary choices optimised for specific outcomes, potentially introducing biases. The authors should better explain the rationale behind their choices to mitigate these concerns.

We thank the reviewer for this valuable comment. In the revised manuscript, we have clarified the rationale behind the main pre-processing steps to explain why specific choices were made and how they help reduce biases. For example, we now express the rationale behind the choice of pseudotime and Mini-Bulk implementation (Results, *lines 116-132*).

In general, we had a conservative approach and always chose the simplest and most controllable steps for the pre-processing. We agree on the fact that our pipeline might introduce biases, meaning that it might not work well on different datasets. For this reason, to further address concerns about potential pre-processing biases, we analysed IGNITE's performance on a dataset (hPSCs) that allowed us to test different characteristics, as discussed in the previous response.

Specifically, part of the pipeline seems to be built to reproduce a specific expression pattern of 24 genes that some of the authors discovered in a previous paper. Although this prior knowledge could be useful and relevant in this specific system, it could limit the generality of the method. For example, the authors selected approximately 2000 genes based on prior knowledge and used a combination of t-SNE and UMAP for dimensionality reduction (although

the two techniques have a similar goal). This specific combination seems to reproduce the pseudotime alignment the authors were expecting to find, but such prior information might not be available in general. Therefore, feature selection and the methods used to project data need more justification, especially if the goal is to create a general tool applicable across different biological systems.

We appreciate the reviewer's feedback regarding the specificity of our pipeline. Our preprocessing was designed to effectively exploit the inherent information of the used dataset. However, we wanted to test the generalizability of IGNITE by applying it to a new publicly available dataset, human PSCs differentiating towards Definitive Endoderm (doi: 10.1186/s13059-016-1033-x).
We selected this dataset for three main reasons: i) It is well studied and has been previously analyzed for GRN inference by Matsumoto et al. and Pratapa et al. (doi: 10.1038/s41592-019-0690-6). ii) It was generated using the Fluidigm C1 system, a single-cell transcriptomics technique different from the 10X platform used in the mouse dataset. This system captures fewer cells, allowing us to assess IGNITE performance with more limited input data. iii) It represents a biological system distinct from the previously analysed mouse model.

Moreover, for the mouse dataset we focused on a small set of genes that may limit generalizability. This choice was made to study a well-characterised system. For the human data we inferred a GRN composed by 98 selected with an unbiased method based on highly variable genes.
For the dimensionality reduction step, we decided to use PCA to test an algorithm that was not already used with the mouse dataset. In this way we tested the capacity of IGNITE in taking as input data preprocessed with different approaches.
Finally, the 2000 genes in the mouse dataset were selected using hierarchical clustering based on Pearson correlation, as described by Carbognin et al. (2023). This was a data-driven approach easily applicable to any dataset, without the need for any prior knowledge. We have clarified this step in the revised manuscript (see **Sec. 5.1.5**).

Analogously, the clustering seems manually adjusted to match known expression patterns of 24 relevant genes, rather than being the result of an optimised clustering method. Additionally, the clusters overlap with different time points, raising concerns about potential batch effects. These issues should be addressed to strengthen the validity of the method.

We thank the reviewer for raising this concern. We would like to clarify that clustering in the mouse dataset was performed on a large set of 2000 highly variable genes, not on the 24 relevant genes used later for downstream analysis. The expression of known marker genes was only used to validate that the clusters captured biologically meaningful structures, and not to define the clusters themselves. This strategy is standard practice in single-cell transcriptomics, and would be equally applied to other systems (e.g., skin stem cells), where clustering is performed on highly variable genes and known markers from the literature are used solely for validation.

Regarding the reviewer's concern about clusters overlapping with different time points and the potential for batch effects, we do not have predefined expectations about the number of clusters and do not exclude the possibility of clusters containing cells from different time points. This overlap may reflect the asynchrony in cell differentiation, where some cells may appear more similar to those at subsequent stages or at earlier stages, rather than strictly to those at their current stage.

In the additional human dataset used for benchmarking, the GRN nodes were not selected a priori but identified in an unbiased manner, by including all transcription factors significantly differentially expressed across clusters. This design choice strengthens the robustness of our approach and supports its general applicability across datasets.

The claims about the comparison with existing methods should be toned down. While the comparisons are useful and interesting, they might be biased due to the method's fine-tuning for the specific system studied. The claim that the model requires only scRNA-seq data is misleading, as strong prior biological knowledge was used to select, for example, the genes analyzed.

We recognise the reviewer's concerns regarding our comparison with existing methods. In the revised manuscript, we expanded our analyses to address IGNITE generalizability: first, we evaluated how the method scales with different numbers of genes and of cells; second, we applied IGNITE to an independent dataset from human PSC differentiation, previously analysed with SCODE. This benchmark allowed us to test the method in a distinct biological system, with fewer cells and a different platform, supporting IGNITE's robustness across contexts.

Regarding the concerns about the need of prior biological knowledge, we stress that the 24-gene network used in the mouse dataset was chosen to facilitate validation in a well-studied system, but it is not a requirement of the method. To demonstrate this, in the human dataset the GRN nodes were identified in an unbiased way. This analysis shows that IGNITE can be applied without relying on prior knowledge, further supporting its generalizability.

Reviewer #3 (Significance (Required)):

The manuscript is scientifically sound, clearly written, and deserves publication. The proposed method is quantitative, novel, theoretically grounded, and was tested in detail with appropriate null models and statistical methods. Moreover, IGNITE can be applied to various biological systems as the availability of scRNA-seq datasets is continuously growing. The paper will be of interest to a broad community of computational biologists and biology labs interested in gene regulation using scRNA-seq data.

The limitation, in my opinion, is the method's (particularly the pre-processing pipeline) fine-tuning for the specific biological system tested. Testing IGNITE on another biological system without pre-selected pre-processing steps or detailed biological priors would be more

convincing and make the paper's conclusions much stronger. The comparison with other methods also may be slightly biased due to this fine-tuning.

My background is in statistical physics, with expertise in biological physics, specifically in mathematical modeling and data analysis in molecular biology.

4. Description of analyses that authors prefer not to carry out

Please include a point-by-point response explaining why some of the requested data or additional analyses might not be necessary or cannot be provided within the scope of a revision. This can be due to time or resource limitations or in case of disagreement about the necessity of such additional data given the scope of the study. Please leave empty if not applicable.

Reviewer #2
When comparing against the ground truth network, the fraction of correctly inferred interactions is technically the same as precision but is ignoring recall. I suggest the authors compute precision, recall and a combined F1 score to compare the evaluated methods.

We thank the reviewer for this thoughtful comment. The ground truth network we used is based on the combination of different experimental data. We first used ChIP-seq, which detects the presence of protein X on the promoter of a target gene Y. Then we used gene expression data (e.g. RNA-seq) generated upon inactivation of protein X and asked whether the expression of gene Y was affected. By combining the two types of data we obtained a list of “true” interactions, those in which X directly regulates the expression of Y.
However, both techniques are prone to false negatives. So we might have failed to detect an interaction, but it does not mean that such interaction does not exist. It is more challenging to establish the absence of a given interaction.
Computing recall and F1 scores would require a comprehensive list of both present and absent interactions, which is currently not available. For this reason, we employed the Fraction of Correctly Inferred Interactions (FCI), which evaluates how well methods recover the known interactions, including their regulatory sign (activation or repression).

Dictys (PMID: 37537351) is a known GRN inference method that also can simulate gene expression but is missing in the benchmark, the authors should add it to the method comparison.

Dictys is a method requiring multi-omics data as input. At least, scRNA-seq and single-cell ATAC-seq are needed. Our experimental data from PSCs could not be analysed with Dictys, as we do not have matching single-cell ATAC-seq data.
Furthermore, as acknowledged by all reviewers, a strength of IGNITE is that it requires only scRNA-seq as input. Thus, a comparison with Dictys would be not informative.

Assuming that Dictys performs better when single-cell multi-omics data are available, IGNITE would still be the preferred choice for most biological systems, for which only scRNA-seq is available.

To make CellOracle results easier to interpret and more comparable, authors should run it at the atlas level instead of at the cell type level, this way generating only one GRN. This can be achieved by assigning the same cluster label to all cells.

The authors of CellOracle recommend generating a distinct GRN for each cell cluster. Predictions are then generated exploiting all GRNs. This should be a strength of their method. For this reason, we think that using the method as recommended by the authors is the fairest way to compare it against IGNITE.
There are several other parameters that differ between CellOracle and IGNITE (e.g. using imputation to deal with zero inflation rather than calculating a Mini-Bulk) and testing all possible combinations of parameters is beyond the scope of our current study.
