# Supplementary material for: Unveiling gene perturbation effects through gene regulatory networks inference from single-cell transcriptomic data
Source: PLoS Comput Biol. 2026 Apr 15;22(4):e1014067. doi: 10.1371/journal.pcbi.1014067 (PMC13082667; doi:10.1371/journal.pcbi.1014067)
Supplement: Attachment — Submitted filename: corridori-et-al-review.pdf [file pcbi.1014067.s010.pdf]

Summary

Corridori and colleagues developed IGNITE, a method to predict gene regulatory networks (GRN) from unperturbed single cell RNA-seq (scRNA-seq) data. Importantly, authors report that their method can efficiently simulate effects from single and multiple gene knockouts. Authors validate their model with two datasets from mouse and human pluripotent stem cells (PSC, mPSC and hPSC, respectively) generated with different sequencing technologies. Authors perform four benchmark tasks on mPSC data. First, they benchmarked the tool against SCODE on the task of modeling unperturbed data. They report comparable capabilities to learn such data. The second task was to compare ability to simulate gene KO across tools. Authors compared IGNITE to SCODE and CellOracle. IGNITE was reported to outperform the other two methods. Importantly, authors note that IGNITE seemed to pick up on features about specific gene activity observed in the triple KO experimental system. The third benchmark task was to compare predicted GRN interactions with publicly available ones. IGNITE predicted interactions were compared to SCODE, CellOracle and MaxEnt. IGNITE recovered two thirds of the known interactions and was outperformed by MaxEnt. The fourth task was about importance of prior knowledge in GRN modeling. Authors report that IGNITE does not simply recover previously discovered interactions but rather learns effective correlations from data. The final sections describe the hPSC dataset, which confirms IGNITE's generalizability across species, sequencing platforms, and biological systems.

Feedback

The manuscript is well written, findings are well supported and clearly described. The paper deserves publication after some revision. The most general comment I have regards the results section. I find some paragraphs (for example 3.4, but others as well) introducing their results in a qualitative manner. I appreciate these descriptions are followed by quantitative statements but a more rigorous presentation would improve delivery and effectiveness of the message. In multiple sections results are presented in a qualitative way followed by a more rigorous quantitative description. I find this redundant.

For example, for section 3.4, I would rewrite "We observed a good agreement between IGNITE simulations and experimental scaled KO–WT differences, with consistent upregulation of naïve genes and downregulation of formative genes in Rbpj, Etv5, and triple KO conditions (Fig. 3A–B). In contrast, SCODE simulations failed to reproduce these gene-level perturbation effects, often showing attenuated or reversed responses. CellOracle captured some expected trends, but with lower fidelity than IGNITE."

into

"IGNITE simulations showed significant correlation with experimental KO–WT differences for Rbpj ($\rho = 0.531$, $p = 9.19 \times 10^{-3}$), Etv5 ($\rho = 0.803$, $p = 3.92 \times 10^{-6}$), and triple KO ($\rho = 0.716$, $p = 2.65 \times 10^{-4}$) conditions (Fig. 3A–B, Table 2). In contrast, SCODE showed weak correlations (ρ = -0.46 to 0.11), while CellOracle showed moderate correlations (ρ = 0.35-0.57)."

This removes subjective language ("good agreement," "failed," "lower fidelity") and lets the quantitative results speak for themselves. Qualitative interpretations like "failed to reproduce" belong in the Discussion, not Results. This applies to multiple sections throughout the manuscript.

Major Comments

1. Results presentation needs to be more rigorous and quantitative

Overall presentation: A general qualitative introduction of the results is given throughout. I appreciate calculation of correlation values with significance testing. However, authors should systematically rephrase sections that qualitatively describe with more rigorous language and presenting the numbers first. A few examples**:**

- Line 217: "good agreement" is redundant when followed by specific Spearman correlations
- Line 220-221: "some expected trends, but with lower fidelity" is vague—the correlation values already convey this
- Line 256: "did not exhibit the expected upregulation" mixes interpretation with observation

I would suggest to lead with quantitative findings. State observations with numbers. Let statistics demonstrate significance. The data in Tables 2-3 are good and support IGNITE's superiority with respect to other tools.

2. Dimensionality reduction approach requires justification

scRNA-seq workflow (mouse data): The use of t-SNE followed by UMAP for dimensionality reduction raises methodological concerns and did it already in the Review Commons process. Both methods fundamentally distort global structure and are typically reserved for visualization purposes only. Authors perform clustering and pseudotime calculation in UMAP space, but distances in UMAP space do not preserve the original relationships in the data.

Standard practice is to perform Principal Component Analysis (PCA) or Canonical Correlation Analysis (CCA) for dimensionality reduction, as these preserve distance

relationships. Methods like the Elbow method or Jackstraw can select the number of components explaining sufficient variance (typically 80%). Clustering and pseudotime are then computed on PCA/CCA-transformed data, with UMAP/t-SNE applied only for final visualization.

I note that authors did use PCA for the hPSC dataset (Fig. S5A). Could authors either:

1. Justify why PCA was unsuitable for mPSC data (e.g., show variance explained plot demonstrating PCA captured insufficient variance), or
2. Apply PCA consistently across both datasets?

If authors have dataset-specific reasons for using t-SNE/UMAP (e.g., linear trajectory with dense sampling), these should be explicitly stated in Methods with supporting evidence. Figure R4 in the response letter suggests PCA explained low variance, but this analysis should be in the manuscript itself.

3. Human PSC section could be better integrated

Overall coherence: While the hPSC analysis includes parallel benchmarks to mPSC (GRN inference, WT generation, KO predictions), the presentation makes it feel like an appendix rather than a core validation. The multi-task benchmark structure applied to mPSC data is less apparent in the hPSC sections (3.7-3.9). My suggestions include:

- Add explicit subsection headings that mirror the mPSC structure (e.g., "3.7 Human GRN Inference and WT Data Generation," "3.8 Evaluating Human GRN Against Known Interactions," "3.9 Human PSC KO Predictions")
- Include a comparative table showing IGNITE performance metrics side-by-side for mPSC and hPSC across all benchmark tasks
- In Discussion, explicitly address what the hPSC results demonstrate about generalizability across species, platforms (10X vs. Fluidigm), and cell numbers (~9500 vs ~750)

This would strengthen the generalizability claims and make clear that hPSC is not just an afterthought but a stringent test of robustness.

Minor Comments

4. Runtime and scalability

Authors mention computational time scales quadratically with gene number (reaching ~12 hours for 200 genes on a laptop). However:

- How does IGNITE runtime compare to SCODE and CellOracle on the same hardware?
- The 500-cell subsampling (Table 6) is informative for cell number scaling, but what about memory requirements?
- A supplementary table showing runtime comparisons would help potential users assess feasibility

5. Code and data availability

I appreciate that code is now available on GitHub (https://github.com/CleliaCorridori/IGNITE). To favor tool adoption and enable larger-scale benchmarking, I have two suggestions.

1. **Code:** Consider packaging as a standalone Python package installable via pip/conda rather than requiring users to clone and navigate Jupyter notebooks. This would significantly lower the barrier to entry.
2. **Data:** While authors state raw scRNA-seq will be available upon publication, making preprocessed data (e.g., the log-normalized matrices with PST and MB) publicly available now would enable immediate reproduction. The European Bioinformatics Institute (EMBL-EBI) provides services to host such data.

6. Minor typographical issues

- Line 451: Missing references for "other studies" validating HNF4A, CXCR4, KLF8, SOX17 roles in endoderm differentiation
- Figure 3E: Y-axis label "Spearman's ρ" would be clearer than "Spearman's p"
- Table 2 caption: Clarify what "statistically significant" threshold means (is it $p < 0.05$ or FDR-corrected?)

Conclusion

This is solid work demonstrating a novel approach to GRN inference and perturbation prediction from unperturbed scRNA-seq data. The core methodology is sound and benchmarking is thorough. My main concern is that Results sections rely too heavily on qualitative descriptions when quantitative results are available and more appropriate. Removing subjective language ("good agreement," "failed," "lower fidelity") and leading with statistics would strengthen scientific rigor. The dimensionality reduction choice also needs better justification or revision. With these changes, the manuscript will be suitable for publication.
