# Supplementary material for: Unveiling gene perturbation effects through gene regulatory networks inference from single-cell transcriptomic data
Source: PLoS Comput Biol. 2026 Apr 15;22(4):e1014067. doi: 10.1371/journal.pcbi.1014067 (PMC13082667; doi:10.1371/journal.pcbi.1014067)
Supplement: Attachment — Submitted filename: Response to reviewers IGNITE 2026.pdf [file pcbi.1014067.s011.pdf]

Response to reviewers

Summary

Corridori and colleagues developed IGNITE, a method to predict gene regulatory networks (GRN) from unperturbed single cell RNA-seq (scRNA-seq) data. Importantly, authors report that their method can efficiently simulate effects from single and multiple gene knockouts. Authors validate their model with two datasets from mouse and human pluripotent stem cells (PSC, mPSC and hPSC, respectively) generated with different sequencing technologies. Authors perform four benchmark tasks on mPSC data. First, they benchmarked the tool against SCODE on the task of modeling unperturbed data. They report comparable capabilities to learn such data. The second task was to compare ability to simulate gene KO across tools. Authors compared IGNITE to SCODE and CellOracle. IGNITE was reported to outperform the other two methods. Importantly, authors note that IGNITE seemed to pick up on features about specific gene activity observed in the triple KO experimental system. The third benchmark task was to compare predicted GRN interactions with publicly available ones. IGNITE predicted interactions were compared to SCODE, CellOracle and MaxEnt. IGNITE recovered two thirds of the known interactions and was outperformed by MaxEnt. The fourth task was about importance of prior knowledge in GRN modeling. Authors report that IGNITE does not simply recover previously discovered interactions but rather learns effective correlations from data. The final sections describe the hPSC dataset, which confirms IGNITE's generalizability across species, sequencing platforms, and biological systems.

We thank the reviewer for this accurate and comprehensive summary of our work, and for the positive assessment of the manuscript. We are pleased that the reviewer recognizes both the methodological contributions of IGNITE and the systematic benchmarking strategy adopted across multiple tasks, datasets, species, and sequencing technologies.
In response to the reviewer's comments, we have revised the manuscript to further clarify methodological aspects, strengthen specific analyses, and improve the presentation and interpretation of our results, as detailed point-by-point below.

Feedback

The manuscript is well written, findings are well supported and clearly described. The paper deserves publication after some revision.
We thank the reviewer for this positive assessment of our manuscript.

The most general comment I have regards the results section. I find some paragraphs (for example 3.4, but others as well) introducing their results in a qualitative manner. I appreciate these descriptions are followed by quantitative statements but a more rigorous presentation would improve delivery and effectiveness of the message. In multiple sections results are presented in a qualitative way followed by a more rigorous quantitative description. I find this redundant.

For example, for section 3.4, I would rewrite "We observed a good agreement between IGNITE simulations and experimental scaled KO–WT differences, with consistent upregulation of naïve genes and downregulation of formative genes in Rbpj, Etv5, and triple

KO conditions (Fig. 3A–B). In contrast, SCODE simulations failed to reproduce these gene-level perturbation effects, often showing attenuated or reversed responses. CellOracle captured some expected trends, but with lower fidelity than IGNITE."
into
"IGNITE simulations showed significant correlation with experimental KO–WT differences for Rbpj ($\rho = 0.531$, $p = 9.19 \times 10^{-3}$), Etv5 ($\rho = 0.803$, $p = 3.92 \times 10^{-6}$), and triple KO ($\rho = 0.716$, $p = 2.65 \times 10^{-4}$) conditions (Fig. 3A–B, Table 2). In contrast, SCODE showed weak correlations (ρ = -0.46 to 0.11), while CellOracle showed moderate correlations (ρ = 0.35-0.57)."

This removes subjective language ("good agreement," "failed," "lower fidelity") and lets the quantitative results speak for themselves. Qualitative interpretations like "failed to reproduce" belong in the Discussion, not Results. This applies to multiple sections throughout the manuscript.

We thank the reviewer for this comment and agree that the Results section benefits from a more quantitative and streamlined presentation. We have revised the Results accordingly, prioritizing quantitative metrics over qualitative descriptors and reducing redundancy across sections. In particular, Section 3.4 was rewritten following the reviewer's suggestion (line 214), introducing results directly through statistical measures. In addition, we applied similar revisions throughout the Results section to reduce qualitative phrasing and evaluative language (e.g. lines 134, 144, 167, 180, 190, 199, 205, 226, 232, 249). These changes emphasize quantitative comparisons and move interpretative statements to the Discussion where appropriate.

Major Comments

1. Results presentation needs to be more rigorous and quantitative

Overall presentation: A general qualitative introduction of the results is given throughout. I appreciate calculation of correlation values with significance testing. However, authors should systematically rephrase sections that qualitatively describe with more rigorous language and presenting the numbers first. A few examples:

- Line 217: "good agreement" is redundant when followed by specific Spearman correlations
- Line 220-221: "some expected trends, but with lower fidelity" is vague—the correlation values already convey this
- Line 256: "did not exhibit the expected upregulation" mixes interpretation with observation

I would suggest to lead with quantitative findings. State observations with numbers. Let statistics demonstrate significance. The data in Tables 2-3 are good and support IGNITE's superiority with respect to other tools.

We thank the reviewer for this comment and agree that the Results section benefits from a more rigorous, quantitative-first presentation. As detailed above, we have revised the Results accordingly. Specifically, qualitative descriptors such as those highlighted by the reviewer were removed or rephrased to report observations in quantitative terms. See lines 214–224 and 242 for the changes implemented following the reviewer's suggestions.

2. Dimensionality reduction approach requires justification

scRNA-seq workflow (mouse data): The use of t-SNE followed by UMAP for dimensionality reduction raises methodological concerns and did it already in the Review Commons process. Both methods fundamentally distort global structure and are typically reserved for visualization purposes only. Authors perform clustering and pseudotime calculation in UMAP space, but distances in UMAP space do not preserve the original relationships in the data.

Standard practice is to perform Principal Component Analysis (PCA) or Canonical Correlation Analysis (CCA) for dimensionality reduction, as these preserve distance relationships. Methods like the Elbow method or Jackstraw can select the number of components explaining sufficient variance (typically 80%). Clustering and pseudotime are then computed on PCA/CCA-transformed data, with UMAP/t-SNE applied only for final visualization.

I note that authors did use PCA for the hPSC dataset (Fig. S5A). Could authors either:

1. Justify why PCA was unsuitable for mPSC data (e.g., show variance explained plot demonstrating PCA captured insufficient variance), or
2. Apply PCA consistently across both datasets?

If authors have dataset-specific reasons for using t-SNE/UMAP (e.g., linear trajectory with dense sampling), these should be explicitly stated in Methods with supporting evidence. Figure R4 in the response letter suggests PCA explained low variance, but this analysis should be in the manuscript itself.

We thank the reviewer for raising this point. As requested, we now explicitly justify in the manuscript why PCA was not adopted for the mouse PSC dataset. All dimensionality reduction steps were evaluated using the same input gene set, and PCA was initially tested. However, PCA captured a limited fraction of the variance and did not clearly separate cells sampled at different time points, consistent with the dense sampling and approximately linear structure of the differentiation trajectory (section 5.1.5, Supplementary Fig. SxA–B).

For this reason, we adopted a nonlinear strategy based on t-SNE followed by UMAP, which more effectively captured local neighborhood structure and preserved temporal ordering in this dataset (https://www.frontiersin.org/journals/genetics/articles/10.3389/fgene.2021.646936/full). This rationale has been added to the Methods section, together with the supporting PCA analysis. By contrast, for the human PSC dataset PCA embeddings were informative and were therefore used for pseudotime inference, as already reported.

3. Human PSC section could be better integrated

Overall coherence: While the hPSC analysis includes parallel benchmarks to mPSC (GRN inference, WT generation, KO predictions), the presentation makes it feel like an appendix rather than a core validation. The multi-task benchmark structure applied to mPSC data is less apparent in the hPSC sections (3.7-3.9).

My suggestions include:

- Add explicit subsection headings that mirror the mPSC structure (e.g., “3.7 Human GRN Inference and WT Data Generation,” “3.8 Evaluating Human GRN Against Known Interactions,” “3.9 Human PSC KO Predictions”)

The hPSC Results were reorganized by introducing subsection headings that mirror the mPSC benchmark structure as suggested.

- Include a comparative table showing IGNITE performance metrics side-by-side for mPSC and hPSC across all benchmark tasks

We added a comparative summary table S1 that presents, side by side, the benchmark tasks and the corresponding performance metrics used for the mPSC and hPSC analyses, highlighting the use of a common benchmarking framework with dataset-specific measures.

- In Discussion, explicitly address what the hPSC results demonstrate about generalizability across species, platforms (10X vs. Fluidigm), and cell numbers (~9500 vs ~750)

This would strengthen the generalizability claims and make clear that hPSC is not just an afterthought but a stringent test of robustness.

In the Discussion, we clarified the role of the hPSC analysis in assessing generalizability across species and experimental platforms (line 486), and revised the corresponding summary statement to make this interpretation explicit (lines 501–504).

Minor Comments

4. Runtime and scalability

Authors mention computational time scales quadratically with gene number (reaching ~12 hours for 200 genes on a laptop). However:

- How does IGNITE runtime compare to SCODE and CellOracle on the same hardware?
- The 500-cell subsampling (Table 6) is informative for cell number scaling, but what about memory requirements?
- A supplementary table showing runtime comparisons would help potential users assess feasibility

We thank the reviewer for raising these practical considerations regarding computational performance.

A direct comparison of runtime and memory usage between IGNITE, SCODE, and CellOracle on identical hardware is outside the scope of this manuscript, as the primary goal of the comparison is to assess the ability of the methods to predict GRN perturbation effects rather than their computational efficiency. The computational performance of SCODE has already been systematically benchmarked in large comparative studies (e.g., Pratapa et al., 2020). CellOracle, on the other hand, relies on precomputed GRNs and rule-based propagation of perturbations, and its original publication does not report detailed analyses of runtime or memory scaling.

In this work, computational performance analyses were included to demonstrate that IGNITE is usable on standard hardware. All reported benchmarks were performed on a standard laptop (MacBook Pro with an Apple M1 Pro processor and 16 GB of RAM). We showed that the computational time scales quadratically with GRN size (quadratic fit $R^2 = 0.9997$), reaching approximately 12 hours for the largest GRN considered, which we consider an acceptable computational cost for the scope of the method.

Regarding memory requirements varying the number of cells, the full mPSC dataset (~10,000 cells) can be processed on a standard laptop (MacBook Pro with an Apple M1 Pro processor and 16 GB of RAM) without exceeding available memory, indicating that memory usage is not a limiting factor at this scale. Subsampling the input to 500 cells further reduces memory usage, while preserving KO-prediction accuracy (Table 6). This indicates that IGNITE is not memory-limited for typical scRNA-seq dataset sizes and remains feasible on standard hardware.

5. Code and data availability

I appreciate that code is now available on GitHub (https://github.com/CleliaCorridori/IGNITE). To favor tool adoption and enable larger-scale benchmarking, I have two suggestions.

1. **Code**: Consider packaging as a standalone Python package installable via pip/conda rather than requiring users to clone and navigate Jupyter notebooks. This would significantly lower the barrier to entry.
   We thank the reviewer for this helpful suggestion. We agree that packaging IGNITE as a standalone Python package would facilitate adoption and large-scale benchmarking. At the current stage, we provide a fully documented GitHub repository containing all scripts and notebooks required to reproduce the results in the manuscript, prioritizing transparency and reproducibility. Packaging as an installable library will be considered in future developments.
2. **Data**: While authors state raw scRNA-seq will be available upon publication, making preprocessed data (e.g., the log-normalized matrices with PST and MB) publicly available now would enable immediate reproduction. The European Bioinformatics Institute (EMBL-EBI) provides services to host such data.
   We thank the reviewer for this helpful suggestion. The preprocessed datasets for both mouse and human PSC analyses are now provided in the GitHub repository to enable immediate reproducibility. Moreover, the mouse raw scRNA-seq data are available through ENA under project ID PRJEB74673.

6. Minor typographical issues

- Line 451: Missing references for "other studies" validating HNF4A, CXCR4, KLF8, SOX17 roles in endoderm differentiation
  We have revised the manuscript to include appropriate references
- Figure 3E: Y-axis label "Spearman's ρ" would be clearer than "Spearman's p"
  We thank the reviewer for the comment. In the submitted version, the y-axis already reports Spearman's correlation coefficient (ρ), not a p-value.
- Table 2 caption: Clarify what "statistically significant" threshold means (is it $p < 0.05$ or FDR-corrected?)
  We thank the reviewer for the suggestion. The significance threshold ($p < 0.05$) is defined in the caption of Table 2 and those are all p values, not FDRs.

Conclusion

This is solid work demonstrating a novel approach to GRN inference and perturbation prediction from unperturbed scRNA-seq data. The core methodology is sound and benchmarking is thorough. My main concern is that Results sections rely too heavily on qualitative descriptions when quantitative results are available and more appropriate.

Removing subjective language (“good agreement,” “failed,” “lower fidelity”) and leading with statistics would strengthen scientific rigor. The dimensionality reduction choice also needs better justification or revision. With these changes, the manuscript will be suitable for publication.

We thank the reviewer for their thorough work on our manuscript. We believe that thanks to their suggestions and comments the work is strongly improved.
